# Uncovering the Optimal Molecular Characteristics of Hydrophobe-Containing Polypeptoids to Induce Liposome or Cell Membrane Fragmentation

**DOI:** 10.1021/acs.biomac.3c00028

**Published:** 2023-02-21

**Authors:** Tianyi Yu, Marzhana Omarova, Meng Zhang, Istiak Hossain, Jianqiang Chen, Omead Darvish, Vijay T. John, Donghui Zhang

**Affiliations:** † Department of Chemistry and Macromolecular Studies Group, Louisiana State University, Baton Rouge, Louisiana 70803, United States; ‡ Department of Chemical and Biomolecular Engineering, Tulane University, New Orleans, Louisiana 70118, United States

## Abstract

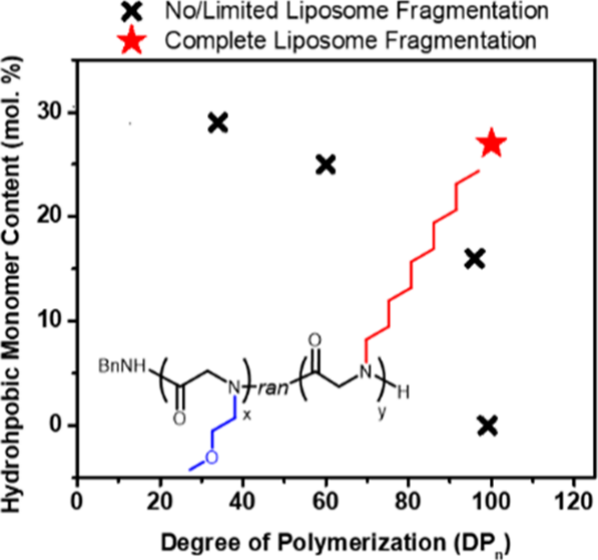

Cellular functions of membrane proteins are strongly coupled to their structures and aggregation states in the cellular membrane. Molecular agents that can induce the fragmentation of lipid membranes are highly sought after as they are potentially useful for extracting membrane proteins in their native lipid environment. Toward this goal, we investigated the fragmentation of synthetic liposome using hydrophobe-containing polypeptoids (HCPs), a class of facially amphiphilic pseudo-peptidic polymers. A series of HCPs with varying chain lengths and hydrophobicities have been designed and synthesized. The effects of polymer molecular characteristics on liposome fragmentation are systemically investigated by a combination of light scattering (SLS/DLS) and transmission electron microscopy (cryo-TEM and negative stained TEM) methods. We demonstrate that HCPs with a sufficient chain length (DP_n_ ≈ 100) and intermediate hydrophobicity (PNDG mol % = 27%) can most effectively induce the fragmentation of liposomes into colloidally stable nanoscale HCP–lipid complexes owing to the high density of local hydrophobic contact between the HCP polymers and lipid membranes. The HCPs can also effectively induce the fragmentation of bacterial lipid-derived liposomes and erythrocyte ghost cells (i.e., empty erythrocytes) to form nanostructures, highlighting the potential of HCPs as novel macromolecular surfactants toward the application of membrane protein extraction.

## Introduction

Approximately 30% of proteins in eukaryotic cells are membrane proteins serving diverse biological functions (e.g., transporters, receptors, anchors, etc.). The structure of membrane proteins is critical for understanding their cellular functions which are associated with many diseases, for example, cystic fibrosis, obesity, and so forth. Membrane proteins represent over 60% of the drug targets,^1^ whereas until 2015, structures of only less than 2% membrane proteins were reported,^2,3^ which strongly limits our understanding of cellular membrane biology and thus the development of drugs targeted on membrane proteins. The major challenge for membrane protein characterization is the intrinsic high hydrophobicity of membrane proteins and the requirement of native lipids environment for their functional stability.

Traditional small-molecule detergents used to isolate membrane proteins can often induce protein aggregation and denaturing.^4,5^ Many efforts have been dedicated to designing new membrane solubilizing agents with enhanced structural stability of encapsulated membrane proteins. Amphipols are short amphiphilic polymers capable of keeping individual membrane proteins soluble in the form of small complexes. Several types of amphipols have been designed and synthesized to replace traditional detergents in stabilizing membrane proteins, for example, A8-35,^6^ glucose-based amphipols,^7^ and so forth. The functional states (e.g., folding structures and oligomeric states) of membrane proteins are better retained in amphipols with enhanced long-term stability as compared to those in detergent-based micelles. However, the functional states of membrane proteins are still different from their initial states in the native cell membranes. As the structure and biological function of membrane proteins are often coupled to their native lipid environment, there is a strong impetus to develop new membrane mimetic platforms for studying and analyzing the structural and functional properties of membrane proteins. Sligar and co-workers developed genetically engineered apolipoprotein, *a*.*k*.*a*. membrane scaffold proteins (MSPs), using apolipoprotein A–I as a model template to produce water soluble MSP-stabilized lipid nanodiscs, which is a lipid patch encircled and stabilized with two belts of helical MSP proteins,^8−12^ and the size homogeneity can be well controlled by optimizing the MSP sequence and the stoichiometry between phospholipids, MSPs, and lipid solubilizing detergents.^12^ Relative to detergent-solubilized membrane protein micelles, MSP lipid nanodiscs provide a better mimetic of the native lipid environment of target membrane proteins, which is beneficial to maintain its native structures and biological functions relative to traditional detergents. However, in the reconstitution of membrane protein embedded MSP-nanodiscs, the choice of detergents used for protein solubilization needs to be optimized based on the specific structures and compositions of target membrane proteins.^10,13,14^ Moreover, the size of MSP-nanodiscs is generally small, with a diameter of ∼10 nm, which are not suitable for large size proteins or protein–protein complexes. Ramamoorthy and co-workers synthesized styrene maleic anhydride (SMA) and SMA derivative polymers which enable the detergent-free extraction of lipids and membrane proteins from both natural and artificial phosphorous lipid bilayers at neutral pH.^15^ Kopf et al. recently found that the installation of appropriate functional groups on the alternating SMA copolymer backbone and side chain can modulate the efficiency of membrane protein solubilization and stability.^16^ Since the aromatic rings in SMA have strong UV absorption which interferes with the characterization of the target membrane protein, styrene-free copolymers, for example, maleic acid and diisobutylene alternating copolymer,^17^ butyl methacrylate and cationic methacroylcholine chloride block copolymers,^18^ alkyl polyacrylic acid copolymers,^19^ and hydrophobically functionalized oligosaccharides/inulin polymers,^20^ have also been synthesized and investigated in the formation of polymer-stabilized lipid nanodiscs. Various membrane proteins such as SecYEG peptide translocon complex,^21,22^ receptor tyrosine kinase epidermal growth factor receptor,^23^ bacteriorhodopsin,^8^ G-protein coupled receptors,^24^ cytochrome P450,^3,25,26^ and so forth, have been successfully reconstituted using MSP or synthetic polymer-stabilized lipid nanodiscs.

For most of the synthetic polymer-stabilized nanodiscs discussed so far, the high hydrophobicity of the fully hydrocarbon-based polymer backbone limits the polymer compositional range that enables the formation of colloidally stable nanodiscs in aqueous solutions. There is a clear need for further development of water-soluble amphiphilic polymers that can stabilize lipid nanodiscs. Polypeptoid, a structural mimic of α-polypeptide, is a facially amphiphilic polymer with a polar polyamide backbone and *N*-substitution with a tunable hydrophilicity–lipophilicity balance. The highly polar backbones can enhance the water solubility of the polypeptoid polymers in a broader compositional range relative to the traditional nanodisc-stabilizing polymers with a hydrocarbon backbone. In addition, without extensive hydrogen bonding along the backbone, the polypeptoid backbone is more flexible relative to other facially amphiphilic detergents (e.g., steroid-based amphiphiles,^6^ short peptides,^27^ etc.), and the polypeptoid backbone conformation can be tailored by the *N*-substituent structure. In addition, synthetic methods to produce well-defined polypeptoids with a range of *N*-substituent structures have been well established.^28,29^ Previous studies by Zuckermann and co-workers have shown that polypeptoids with an optimized sequence and *N*-substituent structures can adopt an extended conformation at oil/water interfaces.^30,31^ Servoss and co-workers have demonstrated that facially amphiphilic sequence-defined peptoid helices preferentially interact with the edges of phospholipid-based bicelles.^32^ Most recently, an amphiphilic peptoid oligomer with a total of 15 residues arranged in a repeating pattern of two *N*-2-ethylphenyl glycine followed by one *N*-2-ethyl carboxyl glycine unit has been synthesized and used to stabilize lipid nanodiscs containing the Pf1 coat protein. The uniformity of the resulting peptoid-stabilized nanodiscs facilitates the solid-state NMR characterization of the trans-membrane protein by enhancing the stability and alignment of nanodiscs under the magnetic field.^33^ As a result, we hypothesized that poly[(*N*-methoxyethyl glycine)-*ran*-(*N*-decyl glycine)] random copolypeptoids [*a*.*k*.*a*. hydrophobe-containing polypeptoids (HCPs)] upon interacting with lipid membranes may adopt an extended polymer conformation with enhanced facial amphiphilicity, thus favoring the formation of polymer belted lipid nanodiscs analogously to the twisted α-helical MSP. We have recently shown that poly[(*N*-methoxyethyl glycine)_76_-*ran*-(*N*-decyl glycine)_24_ (M_76_D_24_) random copolypeptoid can induce the fragmentation of synthetic liposomes to form nanoscale polymer–lipid complexes.^34^ The resulting polymer–lipid complexes not only can modulate the fusogenesis of unilamellar liposomes to form multilamellar liposome; they can also be used to load hydrophobic therapeutics to the liposome.^34,35^ However, it is unclear how the molecular characteristic of HCPs can be adjusted to optimize their efficacy in inducing liposome fragmentation.

In this article, we synthesized a series of HCP copolymers with varying hydrophobicities and molecular weights and investigated their efficacy in inducing the liposome fragmentation in an aqueous solution by a combination of light scattering [static light scattering (SLS)/dynamic light scattering (DLS)] and transmission electron microscopy (cryo-TEM or negative stained TEM) analyses. It was found that HCP copolymers with intermediate hydrophobicity (PNDG mol % = 27%) and sufficient chain length (DP_n_ ≈ 100) can most effectively disrupt synthetic liposomes, bacterial lipid-derived liposomes, and erythrocyte ghosts [i.e., red blood cell (RBC)-derived vesicles], forming nanoscale HCP-lipid fragments that are colloidally stable in aqueous solutions. These results suggest that HCPs are potentially useful as surrogates of surfactants or membrane scaffolding proteins for membrane protein extraction.

## Experimental Section

### Materials

All the chemicals and solvents were purchased from Sigma Aldrich and were used as received unless otherwise noted. The solvents used for polymerization were further purified by using alumina columns under argon protection. CD_2_Cl_2_ and CDCl_3_ were purchased from Cambridge Isotope laboratories. l-α-Phosphatidylcholine (PC) and liposome extrusion setup were purchased from Avanti Polar Lipids. Polycarbonate membranes and membrane filter support were purchased from EMD Millipore. Deionized water used for DLS and SLS was further purified by a Nanopure Bioresearch water purification system with a resistance of 17.8–17.9 MΩ·cm from Barnstead Lab Water Products. ^1^H NMR was collected by a Bruker AV-400 III spectrometer at 298 K and analyzed using Topspin software. Chemical shifts (δ) given in parts per million (ppm) were referenced to protio impurities. *N*-Decyl glycine-derived *N*-carboxyanhydride (De-NCA), *N*-butyl glycine-derived *N*-carboxyanhydride (Bu-NCA), and *N*-methoxyethyl glycine-derived *N-*carboxyanhydride (MeOEt-NCA) monomers were synthesized using a published procedure.^34^

### Synthesis and Characterization of HCP Copolymers

**Scheme 1:**
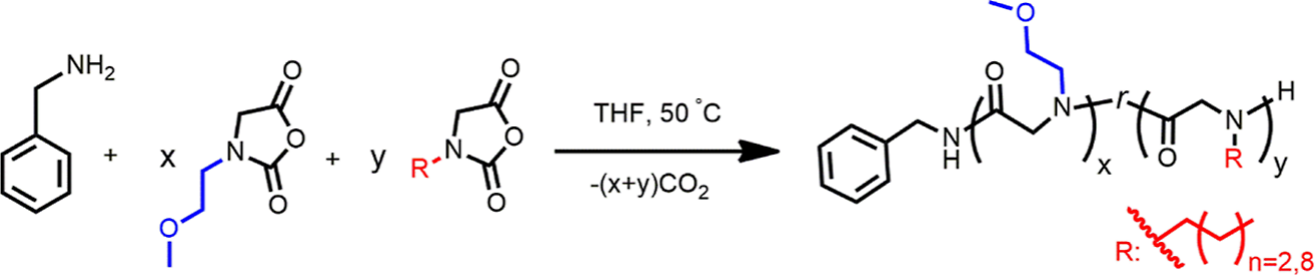
Synthesis of HCP Copolymers

HCP copolypeptoids were synthesized by primary amine-initiated ring-opening polymerization of MeOEt-NCA and De-NCA or Bu-NCA monomers as shown in Scheme 1. A representative procedure for the synthesis of PNMeOEtG_73_-*ran*-PNDG_27_ (HCP-MD100-0.27) is shown. In the glovebox, stock solutions of MeOEt-NCA (*M*_1_, 640 mg, [*M*_1_]_0_ = 0.4 M) and De-NCA (*M*_2_, 968 mg, [*M*_2_]_0_ = 0.4 M) monomers in anhydrous THF were prepared, respectively, in 10 mL volumetric flasks. Benzyl amine stock solution (*I*_0_, 55 mg, [*I*]_0_ = 0.102 M) in anhydrous THF was prepared using a 5 mL volumetric flask. *M*_1_ (2812 μL, 0.18 g, 1.13 mmol, [*M*_1_]_0_ = 0.4 M) and *M*_2_ (939 μL, 0.09 g, 0.39 mmol, [*M*_2_]_0_ = 0.4) stock solutions were fully mixed prior to the addition of initiator stock solution *I*_0_ (148 μL, 0.015 mmol, [*M*_1_ + *M*_2_]_0_: [*I*]_0_ = 100). The polymerization mixture was stirred at 50 °C under nitrogen atmosphere for 96 h to reach complete conversion (note: polymerization time varied depending on different [*M*_1_]_0_:[*M*_2_]_0_ and [*M*_1_ + *M*_2_]_0_:[*I*]_0_ ratios). The polymerization conversion was tracked by monitoring the disappearance of the −C=O peak at 1780 and 1740 cm^–1^ in the reaction aliquots taken over time using FTIR spectroscopy. The volatiles were removed under vacuum using Schlenk line. The crude polymer was further purified by dissolution in DCM and precipitation in hexanes twice. The polymer was isolated by centrifugation and dried under vacuum to yield a white powder (0.19 g, 93%). ^1^H NMR spectrum of PNMeOEtG_73_-*ran*-PNDG_27_ is shown in Figure S1, and ^1^H NMR spectra for other HCP copolymers are shown in Figures S4–S9. ^1^H NMR spectra of PNMeOEtG_73_-*r*-PNDG_27_ (CD_2_Cl_2_, 400 MHz) δ (ppm): 7.23–7.18 (m, C_6_H_5_–, 5H), 4.34–4.02 (m, −COCH_2_−), 3.40–3.19 [m, −CH_2_CH_2_OCH_3_, −NCH_2_– (NDG)], 1.50–1.36 (m, −NCH_2_ CH_2_–, NDG), 1.18 [m, −NCH_2_ CH_2_ (CH_2_)_6_ CH_2_–, NDG], 0.81 (t, −CH_3_, NDG). The polymer compositions were determined by end-group analysis. The number-averaged degree of polymerization (DP_n_) for the PNDG segment (*y* in Scheme 1) was determined by integrating the methyl protons of the PNDG segment centered at 0.81 ppm (*p*, Figure S4) relative to the phenyl protons of the benzyl amine end group at 7.23–7.18 ppm (*r*, Figure S4). The DP_n_ for the PNMeOEtG segment (*x*, Scheme 1) was determined from the integration of −COCH_2_ backbone methylene protons for *x* + *y* at 4.34–4.02 ppm (*b* and *f*, Figure S4) subtracted by the integration of methyl protons at 0.81 ppm for *y*. More specifically, if the integration of end phenyl protons (C_6_H_5_−) was set as 5, DP_n_ (*y*) = integration of CH_3_(p)/3; DP_n_ (*x*) = (integration of ^b,f^–COCH_2_– 2 × *y*)/2, where *p* refers to the end methyl group in the PNDG segment, and *b* and *f* refer to the methylene groups of −COCH_2_ on polymer backbones.

### Size-Exclusion Chromatography

SEC analysis was performed using a Tosoh Bioscience EcoSEC system (HLC 8230 GPC model) equipped with two TSKgel Alpha-M 13 μm, 7.8 mm I.D. × 30 cm columns, a Tosoh Bioscience dual flow RI detector with a 630–670 nm LED light source, and a Tosoh Bioscience LenS3 multiangle light scattering (MALS) detector (30 mW diode laser at λ = 505 nm). SEC experiments were performed in HFIP with 3.0 mg/mL CF_3_CO_2_K at 40 °C with a flow rate of 0.45 mL/min. The HCP polymer solutions were heated at 100 °C for about 10 min until the solutions were visually clear and allowed to stand and cool to room temperature overnight prior to SEC analysis. The solutions were filtered with poly(tetrafluoroethylene) (PTFE) filters (pore size = 0.45 μm) before injecting into the SEC system. The pump housing, column oven, and RI detector temperatures were set at 40 °C. Normalization of the LenS3 detector was conducted using a PMMA standard [*M*_w_ (LS) = 32,350 g/mol, Đ = 1.03] in HFIP/CF_3_CO_2_K (3.0 mg/mL) with a known concentration. All SEC data analysis was performed using SECview software to obtain the polymer *M*_w_, *M*_n_, and Đ.

### Preparation of Liposome Solutions

PC lipids (20 mg) were dissolved in a mixture of chloroform and methanol (5 mL, v/v = 2:1) and gently swirled until the lipid was fully dissolved. The sample was then mixed using rotovap at 25 °C for 10 min with a rotation rate of 120 rpm before turning on the vacuum at 150 mbar for 3 h to slowly remove the volatiles. The vacuum was then increased up to 80 mbar for another 1 h to completely remove all the volatiles, yielding a transparent thin film coated inside of the flask. Nano-pure water (4.0 mL) was added to hydrate the thin film at 50 °C for 2 h with a rotation rate of 80 rpm. The hydration solution was extruded at 50 °C for 20 times with a polycarbonate membrane (pore size = 0.1 μm) to afford a translucent liposome solution (5.0 mg/mL). Further dilution using nano-pure water afforded a liposome solution of 2.5 mg/mL concentration.^35^

### Preparation of Polymer Solutions

A representative procedure for the preparation of the HCP-MD100-0.27 polymer aqueous solution (100 signifies the DP_n_ of the polymer, and 0.27 refers to the molar percentage of the PNDG hydrophobic segment over the entire polymer) at a concentration of 5.0 mg/mL is shown as follows. In a clean scintillation vial, HCP-MD100-0.27 polymer powder (10.0 mg) was dissolved in dichloromethane (DCM, ∼ 1 mL) overnight without agitation. The solution was dried by blowing nitrogen over it to form a transparent polymer thin film, which was hydrated with nano-pure water (2.0 mL). The resultant solution was then stirred at 25 °C overnight with a stirring rate of 350 rpm, yielding a visually clear polymer solution.

### Preparation of Polymer and Liposome Complex (lip + HCP)

A representative procedure for the preparation of HCP-MD100-0.27 polymer and liposome complex (MD100-0.27 + lip) is given as follows. A known volume of HCP-MD100-0.27 polymer solution (5.0 mg/mL) was first filtered through a polyethersulfone (PES) filter (pore size = 0.45 μm) and then added into a freshly extruded liposome aqueous solution (2.5 mg/mL, liposome hydrodynamic diameter = ∼ 0.1 μm) in equal volume (v/v = 1:1). The solution was gently mixed 10 times with a plastic pipette and left to stand at 25 °C for 3 days. The initial translucent solution became clear in 3 days.

### Sample Preparation for Cryo-TEM and Negative Stained TEM Analyses

Cryo-TEM imaging was conducted using an FEI G2 F30 Tecnai TEM operated at 200 kV. The 5.0 mg/mL HCP and 2.5 mg/mL liposome aqueous solutions prepared as described before were mixed in equal volume (v/v = 1:1) and incubated at 25 °C overnight before imaging. 5 μL of the sample solution was transferred to a 200-mesh lacey carbon-coated copper grid (Electron Microscopy Sciences) mounted on the FEI Vitrobot and blotted for 2 s to generate a sample thin film before plunging into liquid ethane.

The morphology of HCPs and the liposome complex was also analyzed using negative stained TEM. The 200-mesh carbon-coated copper grid was pre-treated with a glow-discharger (LEICA EM ACE 600) for 30 s to yield a negatively charged hydrophilic surface. The sample solution (5 μL) was placed on the grid and left for about 5 min before being blotted with a filter paper to remove excess solution. 2 wt % uranyl acetate (5 μL) aqueous solution was subsequently added on the grid and left for 20 s before being blotted using a filter paper.

### Sample Preparation for DLS and SLS Analyses

DLS and SLS measurements were performed on a Wyatt DAWN HELEOS-II instrument with a laser wavelength of 658 nm at 25 °C. The total mean scattered intensity (*I*_90_) (or the count rate) and the normalized second-order autocorrelation function [*g*_2_(*t*)] were recorded at a fixed scattering angle θ = 90° for a total of 300 s with a time interval of 5 and 2 s for the DLS and SLS measurement, respectively.

All the solution samples were passed through a PES syringe filter (pore size = 0.45 μm) prior to SLS and DLS measurements. All the vials, spatulas, and stirring bars used were pre-cleaned with nano-pure water and air-dried before use. Liposome samples as well as liposome and polymer mixtures must be diluted with nano-pure water first before the measurements. For example, 5.0 mg/mL liposome solutions were diluted 40 times prior to the measurement. For HCP and liposome mixtures, every 0.2 mL solution was taken at a controlled time point and diluted 10 times for the measurements. 5.0 mg/mL HCP polymer solutions can be directly measured without any dilution. All solution samples were equilibrated at 25 °C for 5 min before measurement. The DLS exponential decay curve was fitted using maximum entropy method (MEM) analysis to obtain the information of apparent hydrodynamic radius (*R*_h_) and size homogeneity of the samples.

### Determination of Hydrodynamic Radius (*R*_h_)

The correlation function obtained from the DLS measurement was fitted using MEM to obtain the size and distribution information of the apparent hydrodynamic radius (*R*_h,app_).^36,37^ A decay time distribution can be generated using MEM analysis, which was performed using the Clementine (v 1.2) package in Igor Pro (v 6.37) software. The apparent hydrodynamic radius (*R*_h,app_) can be calculated using the Stokes–Einstein equation (eq 2), where *k* is Boltzmann’s constant, *T* is the absolute temperature, η is the solvent viscosity, and *D*_app_ is the apparent diffusion constant which can be calculated using eq 3. In eq 3, *q* is the scattering vector as defined by eq 4, where *n*_0_ is the solvent refraction index, λ_0_ is the vacuum wavelength of the incident light, θ is the scattering angle, and Γ is the decay rate which can be obtained from the correlation function in the DLS measurement (eq 1). The *R*_h,app_ distribution curve can be further fitted with a lognormal distribution function in Origin software to obtain the average *R*_h,app_.
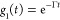
1
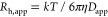
2
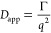
3
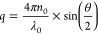
4

### Preparation of Erythrocyte Ghosts

5 mL of bovine whole blood (RBC) was transferred into a 15 mL centrifuge tube. Erythrocytes were separated by centrifugation for 5 min at 500*g*. The supernatant was discarded, leaving packed erythrocytes at the bottom. The centrifugation was repeated three times with phosphate-buffer saline (PBS) solution (3 × 10 mL) to wash off the remaining serum. The resultant packed erythrocytes were subjected to a hypotonic solution (10 mL, PBS solution diluted by 2.5 folds) and incubated for 15 min in an ice bath. Hemoglobin depleted pellets were collected by centrifugation at 15,000*g* for 30 min at 4 °C. The process was repeated until the suspension turned colorless/light pink. Finally, the opaque pellets were resuspended in 5 mL PBS or DI water to obtain micron-sized erythrocyte ghosts.

### Characterizing the Presence of CD47 in the Erythrocyte Ghosts

The RBC-derived ghost suspension (0.3 mL) was incubated with FITC-labeled anti-CD47 antibodies (20 μL) (Santa Cruz Biotechnology, Inc, Dallas, TX) for 1 h at 4 °C. The suspension was then centrifuged (10,000*g*, 10 min) using a centrifuge tube (Amicon Ultra, MWCO 50 kDa) to remove any unbound antibody prior to further analysis with a fluorescence spectrophotometer. As a negative control, PC vesicles (0.3 mL of 0.5% w/v liposomes) were also incubated with FITC-labeled anti-CD47 antibodies and subjected to the same experimental procedure as the RBC-derived ghosts. The fluorescence emission spectra were collected using an excitation wavelength of 465 nm (Figure S23).

## Results and Discussion

### Polymer Synthesis and Characterization

A series of well-defined HCPs, namely, poly[(*N*-methoxyethyl glycine)-*ran*-(*N*-decyl glycine)] (M_*x*_D_*y*_) or poly[(*N*-methoxyethyl glycine)-*ran*-(*N*-butyl glycine)] (M_*x*_B_*y*_), with varying molecular weights and molar fractions of the hydrophobic segments (D or B, Scheme 1), have been synthesized by primary amine-initiated copolymerization of *N*-methoxyethyl glycine-derived *N*-carboxyanhydride (MeOEt-NCA) and *N*-decyl glycine-derived *N*-carboxyanhydride (De-NCA) [or *N*-butyl glycine-derived *N*-carboxyanhydride (Bu-NCA)] monomers by adapting a published procedure.^34^ The molecular structure of the monomers and HCPs has been verified by ^1^H NMR analysis (Figures S1–S9). The polymer molecular weight (*M*_n_) and molecular weight distribution (Đ = *M*_w_/*M*_n_) have been determined by SEC-dRI-MALS analysis of the polymer solution with their respective dn/dc values in HFIP with 3 mg/mL CF_3_CO_2_K and are summarized in Table 1. Three HCP copolymers with a constant molar percentage of hydrophobic segments (PNDG mol % = 27 ± 2%) and varying molecular weights or chain lengths (DP_n_ = 34, 60, and 100), abbreviated as HCP-MD34-0.29, HCP-MD60-0.25, and HCP-MD100-0.27 (entries 2–4, Table 1), were synthesized to enable the investigation of the chain length effect on the efficacy of polymers in causing liposome fragmentation. Three HCP copolymers with a nearly identical chain length (DP_n_ = 95–100) and varying molar fractions of the hydrophobic segment (PNDG mol % = 16 and 27%, PNBG mol % = 25%) abbreviated as HCP-MD96-0.16, HCP-MD100-0.27, and HCP-MB95-0.25 and one non-hydrophobe-containing polypeptoid (NCP) with zero PNDG or PNBG content and similar chain length (DP_n_ = 99) (NCP-M99-0.00) (entries 1, 4–6, Table 1) were synthesized to enable the elucidation of the role of polymer hydrophobicity in inducing liposome fragmentation. Note that we use the molar fraction of the hydrophobic segment in the HCP copolymers as an indicator of the relative hydrophobicity or hydrophobic content of the copolymers.

**Table 1:** Molecular Characteristics of PNMeOEtG_*x*_-*r*-PND(B)G_*y*_ (M_*x*_D(B)_*y*_) Copolymers or PNMeOEtG_*x*_ (M_*x*_) Homopolymers

entry#	sample name^a^	polymer composition^b^	*M*_n_^c^ (kg/mol)	Đ^c^	PND(B)G molar content^a^ (%)
1	HCP-MB95-0.25	M71B24	10.9	1.05	25
2	HCP-MD34-0.29	M24D10	4.7	1.17	29
3	HCP-MD60-0.25	M45D15	8.1	1.11	25
4	HCP-MD100-0.27	M73D27	13.7	1.04	27
5	HCP-MD96-0.16	M81D15	12.3	1.05	16
6	NCP-M99-0.00	M99	11.4	1.09	0

a M signifies the solvophilic PNMeOEtG segment, and B or D represents the solvophobic PNB(D)G segment. The first number in the sample name signifies the total number-average degree of polymerization, and the second number signifies the molar fraction of the solvophobic segment (D or B). PND(B)G % refers to the molar percentage of the PND(B)G segment in the respective HCPs.

b The subscript signifies the number-average degree of polymerization for the respective solvophilic (M) and solvophobic (B or D) segment of HCPs, as determined by ^1^H NMR analysis.

c The number-averaged molecular weight (*M*_n_) and molecular weight distribution (Đ = *M*_w_/*M*_n_) were determined by SEC-dRI-MALS analysis in HFIP/CF_3_CO_2_K (3.0 mg/mL) at 40 °C.

### Characterizations of Liposomes and HCP/NCP in Aqueous Solutions

Synthetic liposomes comprising PC were prepared by a membrane extrusion method and were used as a lipid bilayer membrane model. The preparation details are described in the Experimental Section. The liposome solution was monitored using DLS at room temperature over the course of 3 days, and the normalized autocorrelation function [*G*(*t*)] was analyzed using MEM to obtain the mean hydrodynamic radius (*R*_h_) and the size distribution of the liposomes. DLS analysis of the liposome solution revealed a minor reduction of *R*_h_ from 53.5 ± 0.3 nm to 45.1 ± 0.2 nm after 3 days of incubation at room temperature (Figure S11A,B). As a result, all liposome solutions were freshly prepared and immediately used in the subsequent study of polymer-induced liposome fragmentation.

A series of aqueous solutions of HCP and NCP polymers with varying molecular weights (DP_n_ = 34–100) and hydrophobicities [PND(B)G mol. % = 0–27] were prepared at a polymer concentration of 5 mg/mL in ultrapure water. The solution preparation details are described in the Experimental Section. While all polymer solutions appear visually clear, facially amphiphilic polymers can form molecular aggregates in aqueous solutions.^15^ As a result, we first conducted DLS analysis of these polymer solutions to verify the aggregation state of the polymers in aqueous solutions. DLS analysis by the MEM method revealed two sized populations for all HCP solutions (Figures S12 and S13). The population at short decay time was attributed to individually dissolved polymers with hydrodynamic radius (*R*_h_) in the 2.8 ± 0.4–6.5 ± 0.7 nm range, whereas the population at longer decay time was due to polymer aggregates with *R*_h_ in the 15.9 ± 0.3–78.0 ± 0.3 nm range (Table S1). The scattering intensity ratio of the dissolved polymer over the polymer aggregates (*I*_1_/*I*_2_) is in the range of 0.04–0.004 for varying HCP solutions (Table S1 and Figure S13). As the scattering intensity is strongly dependent on the particle size (*I* ∼ *r*^6^, *r* is particle radius), it is evident that the number abundance of individually dissolved HCP polymers greatly exceeds that of the corresponding polymer aggregates by several order of magnitudes (1.4 × 10^3^–1.5 × 10^5^, Table S1) in water at a polymer concentration of 5 mg/mL. Cryo-TEM analysis of selected HCP polymers (HCP-MD100-0.27 and HCP-MD34-0.29) revealed the presence of mostly spherical particles and occasional elongated particles (Figure S14), consistent with the DLS results.

### Polymer Concentration Effect on Liposome Fragmentation

A series of aqueous solutions of HCP-MD100-0.27 polymer (entry 4, Table 1) with varying polymer concentrations (0.6–5.0 mg/mL) were individually added to freshly prepared liposome solutions (2.5 mg/mL) in equal volume at room temperature. The resulting liposome with HCP-MD100-0.27 solutions, abbreviated as lip + HCP-MD100-0.27, with a constant liposome concentration (1.3 mg/mL) and varying HCP-MD100-0.27 concentrations (0.3, 0.6, 1.3, and 2.5 mg/mL) were each allowed to stand at 25 °C for 3 days. A liposome solution without any HCP-MD100-0.27 served as a control.

**Figure 1:**
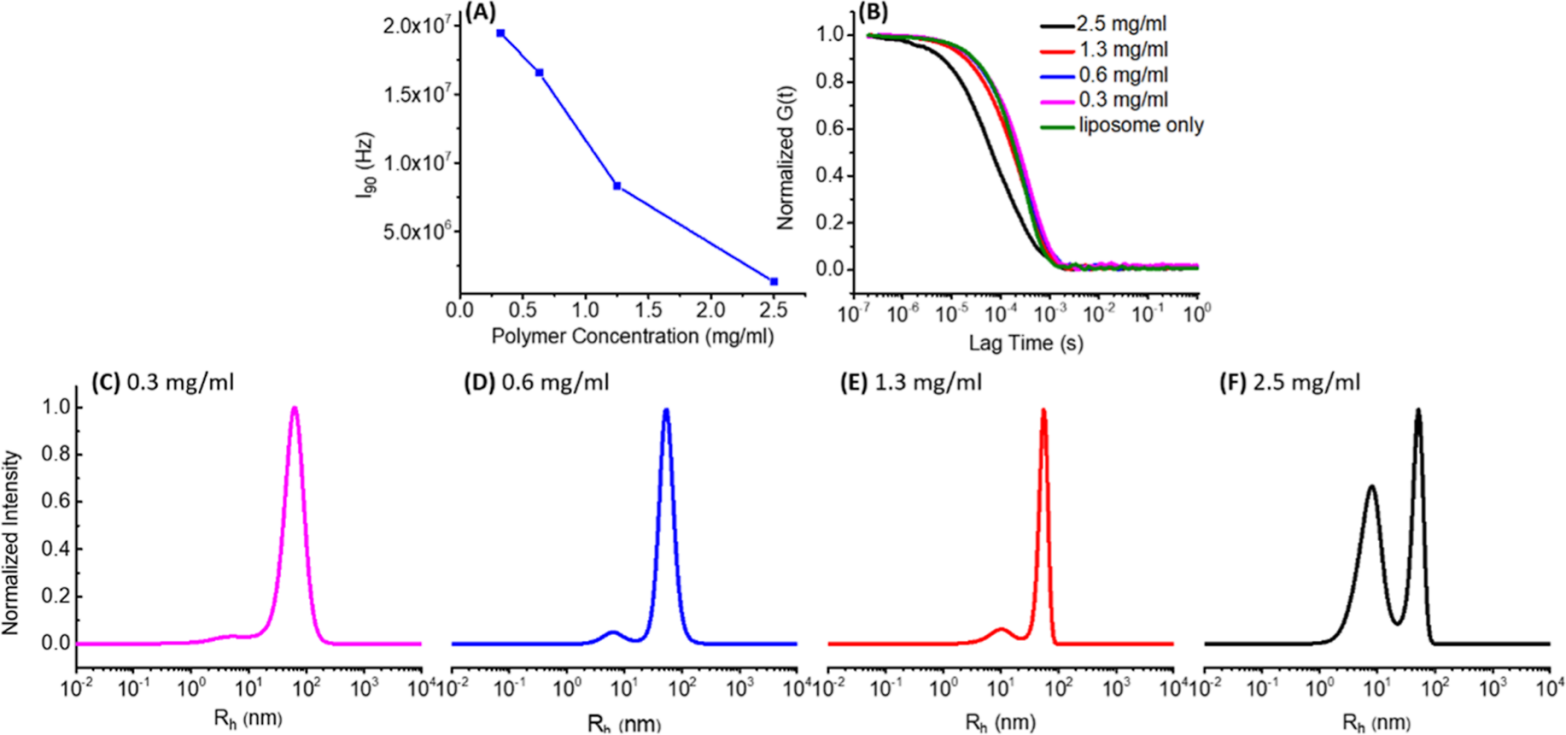
SLS and DLS results showing the effect of the HCP polymer concentration in inducing liposome fragmentation. (A) Plots of the mean SLS scattering intensity (*I*_90_) of the liposomes and HCP-MD100-0.27 solutions (lip + HCP-MD100-0.27) vs the HCP-MD100-0.27 polymer concentration. (B) Normalized autocorrelation function obtained by DLS measurements. (C–F) Intensity-weighted particle size distribution, for the respective liposome and HCP-MD100-0.27 polymer solution with varying HCP-MD100-0.27 concentrations (0.3, 0.6, 1.3, and 2.5 mg/mL) after incubation at room temperature for 3 days.

The initially translucent liposome-HCP-MD100-0.27 polymer solutions were found to become clear without any noticeable precipitates after 3 days, whereas the liposome solution without any added polymer remained translucent for the entire duration. The solution turbidity change over time was characterized by SLS measurements (Figure 1A). The mean count rate measured at an angle of 90°, which represents the mean scattering intensity (*I*_90_), is approximately the same in the 1.8 × 10^7^–2.0 × 10^7^ Hz range for all samples at time = 0. After incubation for 3 days, the mean scattering intensity decreased substantially, and the extent of reduction was nearly proportional with increasing HCP-MD100-0.27 polymer concentrations. For example, *I*_90_ was found to decrease by a factor of 10 at the highest HCP-MD100-0.27 polymer concentration (2.5 mg/mL). By contrast, *I*_90_ remained relatively stable at ∼ 2.0 × 10^7^ Hz for the liposome solution control over the course of 3 days. These results strongly indicate that the HCP-MD100-0.27 polymer can break down liposomes in the aqueous solution and a higher HCP-MD100-0.27 concentration can induce a greater extent of liposome fragmentation.

The liposome-HCP-MD100-0.27 polymer solutions after incubation for 3 days were further characterized by the DLS technique (Figure 1B). The DLS trace, that is, the normalized autocorrelation function [*G*(*t*)] versus the lag time plot, exhibited a notable shift to a lower lag time for the solution with the highest added HCP-MD100-0.27 polymer concentration (2.5 mg/mL) relative to those with lower HCP-MD100-0.27 concentrations (0.3–1.3 mg/mL), consistent with a greater extent of liposome fragmentation at the higher polymer concentration. DLS analysis by the MEM method (Figure 1C–F) revealed the presence of two particle populations. The population at the longer lag time with *R*_h_ in the 52.5 ± 0.2–70.8 ± 0.4 nm range (Table S2) is attributed to the remaining liposomes and possibly large structural assemblies of liposomes and HCP-MD100-0.27 polymers, and the population at the shorter lag time with *R*_h_ in the 7.8 ± 0.4–12.7 ± 0.5 nm range (Table S2) is attributed to the HCP–lipid complexes resulting from the liposome fragmentation. It is evident that the highest polymer concentration (*c*_polymer_ = 2.5 mg/mL) is the most effective in inducing liposome fragmentation, evidenced by the significantly enhanced intensity of the particle population at the short lag time relative to that at the long lag time. Given that the scattering intensity is strongly dependent on the particle size (*I*_90_ ∼ *r*^6^, *r* is particle radius), a relative scattering intensity ratio of 1:4.1 approximately corresponds to a number ratio of 7600:1 for the small versus large particle in the case of the liposome-HCP-MD100-0.27 polymer solution at a polymer concentration of 2.5 mg/mL (Figure 1F). This implies that most of the liposomes have been effectively fragmented by HCP-MD100-0.27 at a polymer concentration of 2.5 mg/mL. Based on the combined SLS and DLS results, 2.5 mg/mL is used as a standard polymer concentration for further study on the polymer-induced liposome fragmentation with other HCP polymers.

### Polymer Chain Length Effect on Liposome Fragmentation

**Figure 2:**
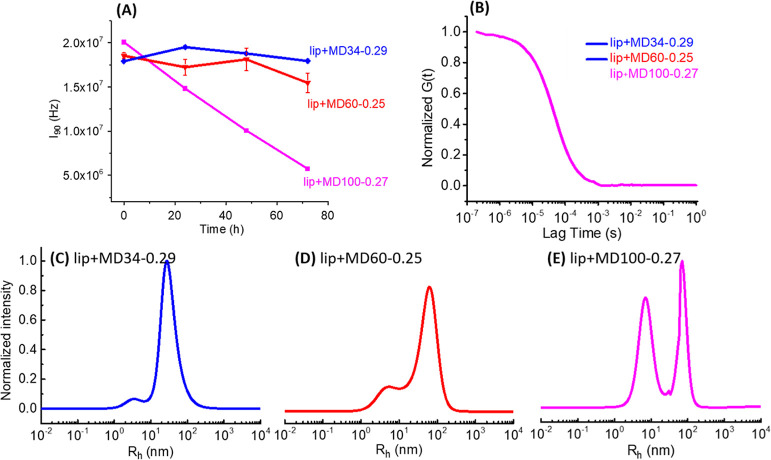
SLS and DLS results showing the effect of the HCP polymer chain length in inducing liposome fragmentation. (A) Plots of the mean SLS scattering intensity (*I*_90_) vs incubation time, (B) normalized autocorrelation function obtained by DLS measurements, and (C–E) intensity-weighted particle size distribution for the respective liposome-HCP polymer solutions containing HCPs with different chain lengths (DP_n_ = 34, 60, and 100), after incubation at room temperature for 3 days.

A series of HCP polymers having a constant fraction of hydrophobic segment (PNDG mol % = 27 ± 2 mol %) and varying chain lengths (DP_n_ = 100, 60, and 34) (entries 2–4, Table 1) have been used to investigate the chain length effect of HCP copolymers in inducing liposome fragmentation. Aqueous solutions containing liposomes (1.3 mg/mL) and respective HCP-MD100-0.27, HCP-MD60-0.25, or HCP-MD34-0.29 polymers (2.5 mg/mL) were allowed to stand at room temperature for 3 days. The initially translucent solutions became clear without any noticeable precipitates after 3 days. SLS analysis revealed that the liposome-HCP-MD100-0.27 polymer solution exhibited the most notable reduction of the total scattering intensity over time, followed by the liposome-HCP-MD60-0.25 polymer solution (Figure 2A). The liposome-HCP-MD34-0.29 polymer solution exhibited a minimal change of total scattering intensity over the same duration. These results indicate that the efficacy of the HCP polymer-induced liposome fragmentation is strongly dependent on the polymer molecular weight or chain length, with the long HCP polymer (DP_n_ = 100) inducing a much faster and greater extent of liposome fragmentation relative to shorter ones (DP_n_ = 60 and 34).

DLS analysis of the liposome-HCP polymer solutions with respective HCP-MD100-0.27, HCP-MD60-0.25, or HCP-MD34-0.29 polymers revealed a systematic reduction of particle size over the course of 3 days, evidenced by the shift of the normalized autocorrelation function to the shorter lag time with prolonged incubation time (Figure S16). At the 3 day time point, the size reduction is most pronounced for the liposome-HCP-MD100-0.27 polymer solution (Figure 2B). In addition, the MEM analysis of DLS data revealed the presence of two particle populations with different sizes for all the samples (Figures 2C–E and S17). The apparent hydrodynamic size (*R*_h_) of the particle population at the short lag time increased from 4.6 ± 0.5 to 6.7 ± 0.4 to 8.6 ± 0.4 nm, and the *R*_h_ at the long lag time increased from 34.7 ± 0.4 to 73.6 ± 0.5 to 77.4 ± 0.2 nm (Table S3), with the increasing chain length of HCP polymers. The population at the short lag time is attributed to HCP–lipid complexes resulting from liposome fragmentation, and the population at the long lag time is presumably due to remaining liposomes or possibly large structural assemblies of liposomes and HCP polymers. Importantly, as the HCP chain length increased, the intensity of the small-sized population relative to that of the large-sized population increased accordingly, strongly indicating that HCPs with longer chain lengths are more effective in inducing liposome fragmentation relative to the shorter ones.

As the short HCP-MD34-0.29 polymer is significantly less effective in inducing liposome fragmentation at a standard concentration of 2.5 mg/mL, higher polymer concentrations (i.e., 5.0 and 7.5 mg/mL) were further investigated in the liposome fragmentation under identical conditions. DLS analysis using the MEM method revealed that tripling the added HCP-MD34-0.29 polymer concentration (7.5 mg/mL) can augment the extent of liposome fragmentation, evidenced by the increased content of small particle population relative to the large one after 3 day incubation (Figures S18 and S19). By contrast, at lower HCP-MD34-0.29 concentrations (2.5–5.0 mg/mL), the liposome remains mostly intact.

While tripling the concentration of the short HCP-MD34-0.29 polymer can induce liposome fragmentation, the final extent of fragmentation at a concentration of 7.5 mg/mL is still lower than that caused by the long HCP-MD100-0.27 polymer at a low concentration of 2.5 mg/mL, evidenced by the lower intensity ratio of the small-sized population relative to the large one for the former compared to the latter (Figures S19 and 2E). It is clear that the short-chain HCP polymers are not as effective in inducing liposome fragmentation relative to the long chain counterparts. This suggests that the higher local density of hydrophobic side chains in long polymers is important for inducing liposome fragmentation, which is consistent with a previous molecular dynamics simulation on SMA polymer-induced fragmentation of lipid membranes.^15^

**Figure 3:**
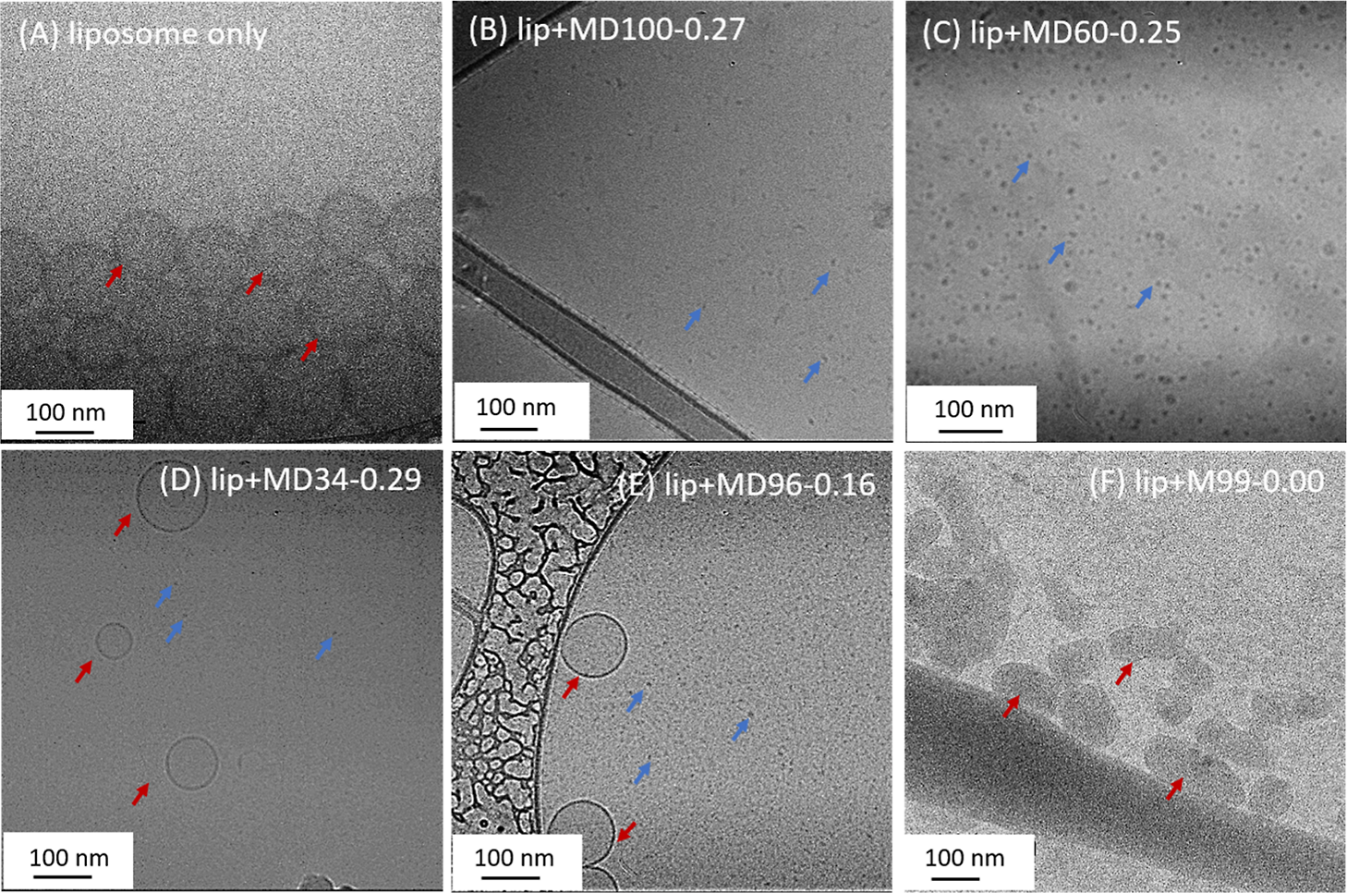
Representative cryo-TEM images of (A) liposome solution, (B) liposome-HCP-MD100-0.27 polymer solution, (C) liposome-HCP-MD60-0.25 polymer solution, (D) liposome-HCP-MD34-0.29 polymer solution, (E) liposome-HCP-MD96-0.16 polymer solution, and (F) liposome-NCP-M99-0.00 polymer solution after incubation at room temperature for 3 days. The HCP–lipid complexes resulting from liposome fragmentation are indicated with blue arrows. Liposomes are indicated by red arrows.

The liposome-HCP-MD100-0.27, liposome-HCP-MD60-0.25, and liposome-HCP-MD34-0.29 polymer solutions after incubation for 3 days were further characterized by the cryo-TEM (Figure 3B–D) and negative stained TEM (Figure S21–F) methods. The liposome solution without the addition of any polymer was also imaged for comparison (Figures 3A and S21A,B). No residual liposomes and only small spherical nanostructures in the ca. 5.6–9.6 nm size range were observed for the liposome-HCP-MD100-0.27 polymer solution that was incubated for 3 days (Figures 3B and S21C,D), supporting that the liposomes were disrupted to form small fragments in the presence of the HCP-MD100-0.27 polymer, in agreement with the DLS/SLS results. By contrast, short fibrils with a contour length in the range of ca. 23.9–36.6 nm and spherical nanostructures with a diameter in the range of ca. 5.0–6.1 nm were found to co-exist with intact liposomes or large lipid-HCP assembles in the liposome-HCP-MD34-0.29 polymer (Figures 3D and S21E,F). Based on the combined TEM and DLS/SLS results, it is evident that long HCP-MD polymers (DP_n_ = 100) can more effectively induce liposome fragmentation with a faster and higher extent of fragmentation relative to shorter ones with DP_n_ = 34 or 60.

### Polymer Hydrophobicity Effect on Liposome Fragmentation

**Figure 4:**
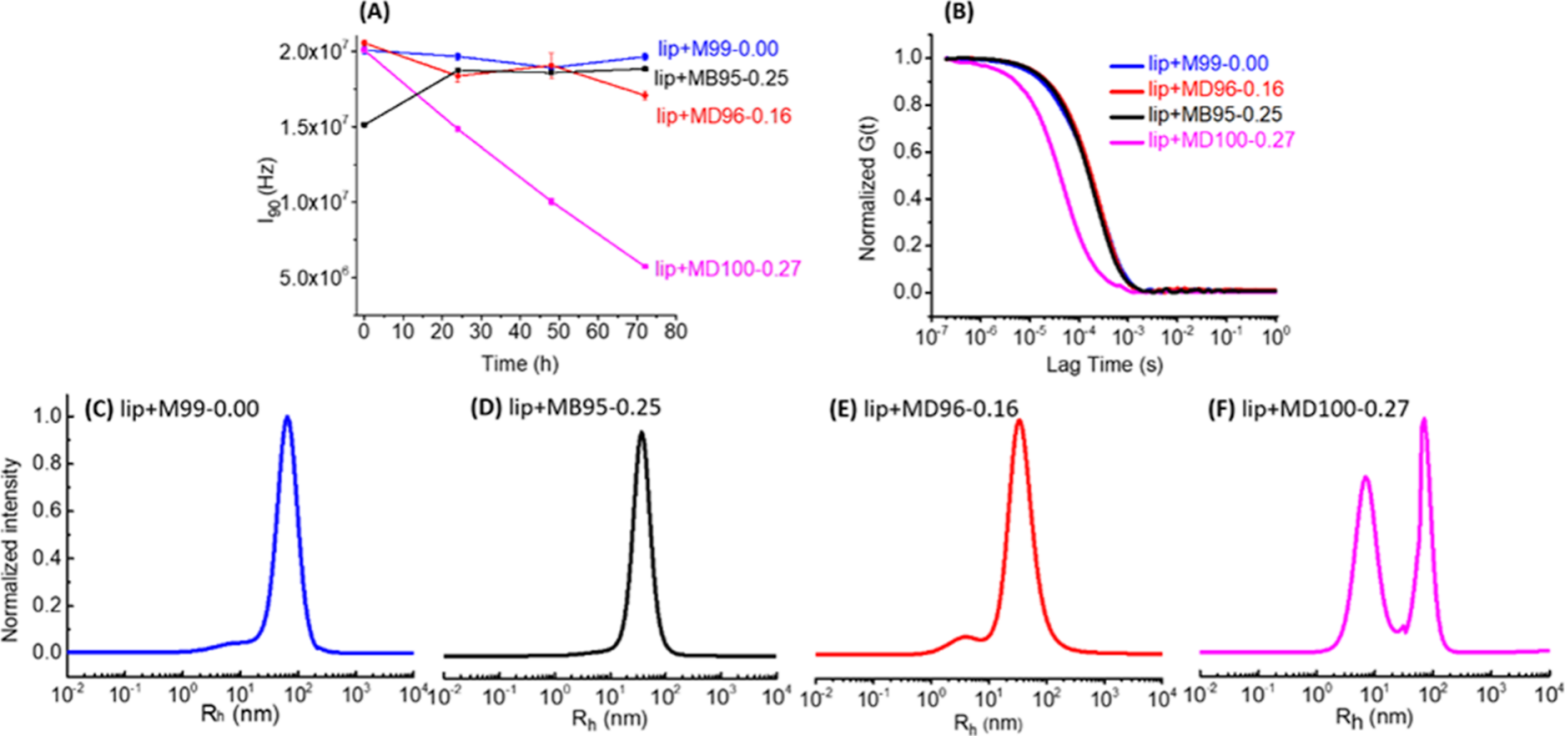
SLS and DLS results showing the effect of HCP hydrophobicity in inducing liposome fragmentation. (A) Plots of mean SLS scattering intensity (*I*_90_) vs incubation time, (B) normalized autocorrelation function obtained by DLS measurements, and (C–F) intensity-weighted particle size distribution, for the respective liposome–HCP polymer or liposome–NCP polymer solutions containing polypeptoid polymers with varying PND(B)G mol % after incubation at room temperature for 3 days.

The effect of polymer hydrophobicity on liposome fragmentation was investigated by comparing the hydrophilic poly(*N*-methoxyethyl glycine) homopolymer PNMeOEt_99_ (NCP-M99–0.00), *a*.*k*.*a*. NCP, with three HCPs having similar chain lengths (DP_n_ = 95, 96, and 100) and varying hydrophobic *N*-decyl or *N*-butyl contents (i.e., PNBG mol % = 25%, PNDG mol % = 27 and 16%). The aqueous solutions containing the liposome (1.3 mg/mL) and these respective NCP or HCP polymers (2.5 mg/mL) were incubated at room temperature for 3 days. The particle size changes in these solutions were monitored using SLS (Figure 4A) and DLS techniques (Figures 4B and S20).

SLS analysis revealed that the total scattered light intensity (*I*_90_) has no significant change for the liposome solution with NCP polymers (lip + NCP-M99–0.00 in Figure 4A) over the course of 3 days. Only a small *I*_90_ reduction from 2.0× 10^7^ to 1.8 × 10^7^ Hz was observed for the liposomes, with HCP having a low content (PNDG mol % = 16%) of the *N*-decyl segment (lip + HCP-MD96-0.16 in Figure 4A). By contrast, a linear and pronounced reduction from 2.0 × 10^7^ to 5.0 × 10^6^ Hz was observed for the liposome-HCP solutions with a high content (PNDG mol % = 27%) of *N*-decyl hydrophobe over the same duration (lip + HCP-MD100-0.27) (Figure 4A). Meanwhile, the DLS measurements revealed a notable shift toward a lower lag time in the normalized autocorrelation function [*G*(*t*)] for the liposomes with the HCP-MD100-0.27 polymer solution over the 3 day period (Figure S20D), whereas no obvious shifts were observed for the liposome solutions with NCP-M99-0.00 and HCP-MD96-0.16 polymer solutions over the same duration (Figure S20A,B). DLS analysis by the MEM method revealed the presence of two particle populations for liposomes with HCP-MD100-0.27 and HCP-MD96-0.16 polymers after incubation for 3 days (Figure 4E,F): a small-sized population with a diameter in the 4.4 ± 0.2–8.6 ± 0.4 nm range and a large-sized population with a diameter in the 43.0 ± 0.5–77.4 ± 0.2 nm range (Table S3). The relative abundance of the small-sized particle relative to the large-sized one decreases as the hydrophobic content (PNDG mol %) of the polymers decreases from 27 to 16 to 0%. The combined SLS and DLS results strongly suggest that HCP polymers with a higher hydrophobic content (i.e., HCP-MD100-0.27) is more effective in inducing liposome fragmentation than those with less hydrophobic content.

To investigate the effect of the chemical nature of the hydrophobic side chains (hydrophobes) on the liposome fragmentation, we compared liposomes with two HCP samples, HCP-MD100-0.27 and HCP-MB95-0.25, having a similar molar content of the hydrophobic segments (PNDG mol % = 27 mol% vs PNBG mol% = 25%) and chain lengths (DP_n_ = 100 and 95) but different hydrophobic side chains (*N*-decyl vs *N*-butyl). SLS analysis in Figure 4A shows a slight increment of *I*_90_ from 1.5 × 10^7^ to 1.9 × 10^7^ Hz for liposomes with HCP-MB95–0.25, whereas a significant reduction from 2.0 × 10^7^ to 5.0 × 10^6^ Hz was observed for liposomes with HCP-MD100-0.27, suggesting a more complete fragmentation in the presence of the HCP-MD100-0.27 polymer. Furthermore, the intensity-weighted particle size distribution in Figure 4D shows only one peak with a diameter of 41.9 ± 0.4 nm for liposome-HCP-MB95-0.25, suggesting no significant liposome fragmentation, in contrast to the nearly complete fragmentation for liposome-HCP-MD100-0.27 (Figure 4F). These combined results indicate that high local density of the hydrophobes is critical in fragmenting lipid membranes.

The solution morphology of liposome and HCP polymer solutions after 3 day incubation was further analyzed by cryo-TEM (Figure 3B,E–F) and negative stained TEM (Figure S21C,D,G–J) methods. While only liposome fragments or lipid–HCP complexes in ca. 5.6–9.6 nm size range was observed for the liposome and HCP-MD100-0.27 solution (Figures 3B and S21C,D), residual liposomes were still notably present in the liposomes with HCP-MD96-0.16 or NCP-M99–0.00 solutions (Figures 3E,F and S21G–J), indicating incomplete liposome fragmentation by less hydrophobic HCPs in accordance with the DLS and SLS results. It is interesting to note that while the least hydrophobic NCP-M99–0.00 polymer failed to induce liposome fragmentation, the initial spherical liposomes were deformed into elongated shapes. This suggests that there may still be interaction between the NCP-M99–0.00 polymer and the liposome (Figures 3F and S21I,J), the nature of which is currently unclear.

The combined experimental results indicate that the density of hydrophobic side chains in the HCP polymer and the polymer chain length are both important molecular characteristics that modulate the interaction between the HCPs and the liposome, resulting in varying levels of efficacy (i.e., the extent and rate of fragmentation) in liposome fragmentation. The higher density of hydrophobic side chains on HCPs can presumably promote membrane deformation and instability by increasing local hydrophobic interactions with the lipid tails in the membrane. In addition, the liposome fragments may be better stabilized by longer chain HCPs than the shorter ones due to reduced entropic penalty associated with the formation of HCP–lipid complexes for the former relative to the latter.^15^

The liposome fragmentation is likely to be initiated by the multiple insertions of the hydrophobic side chains of HCP copolymers into the lipid bilayers, resulting in the local destabilization of the liposome and the formation of lipid bilayer fragments. This is supported by the observation of multi-lamellar liposome structures formed by the attachment of the lipid bilayer fragments to the intact liposomes via HCP when insufficient HCP is present to induce complete liposome fragmentation.^34^ When sufficient HCP is present, the liposomes are fully fragmented to form HCP–lipid complexes.^34^

### HCP-Induced Fragmentation of Bacterial Lipid-Based Liposomes and Erythrocyte Ghost Cells

As the cellular membrane comprising mixed lipids and other biologically relevant molecules is more complex than synthetic liposomes, it is important to investigate whether the HCP polymers can induce the fragmentation of biologically relevant membranes. As such, we investigated whether HCP polymers can induce the fragmentation of liposomes comprising *Escherichia coli* lipid extracts. HCP-MD92-0.22 was mixed with liposomes comprising *E. coli* lipid extracts and incubated at room temperature for 3 days. Cryo-TEM analysis of the solution after incubation for 3 days revealed the complete fragmentation of the *E. coli* lipid-derived liposomes (Figure S22A) into ca. 4.5–6.8 nm sized nanostructures presumably comprising *E. coli* lipids and HCP polymers (Figure S22B).

**Figure 5:**
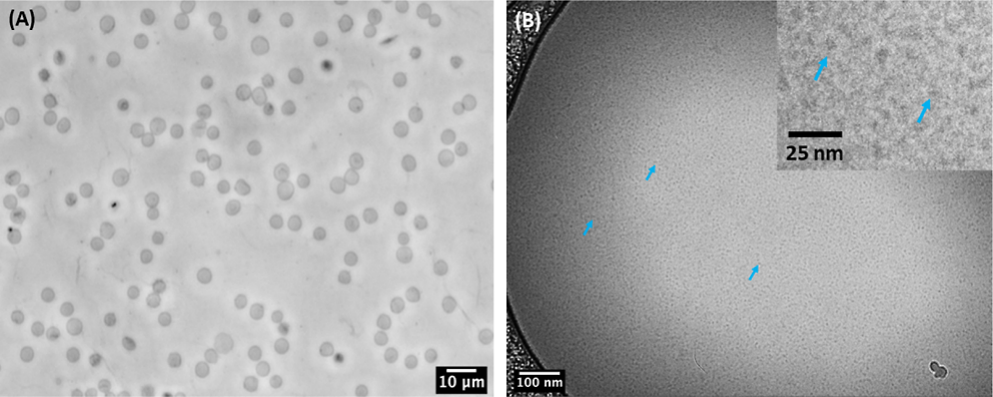
Phase contrast optical micrograph of (A) erythrocyte ghosts in DI water (∼4 μm) and ghost–HCP complexes formed by the HCP-MD100-0.27 polymer, which are too small to be resolved with optical microscopy. Cryo-TEM images of ghost–HCP complexes (B) and an expanded region of the cryo-TEM images (inset). The fragmented ghost-derived HCP complexes (∼8 nm) are indicated with arrows.

Encouraged by this finding, we further investigated whether the HCPs can induce the fragmentation of erythrocyte ghost derived from RBCs. The ghosts are large vesicles comprising an intact RBC membrane with all membrane components (e.g., membrane proteins, native lipids, etc.) present and all intracellular components including hemoglobin removed, which makes ghost a viable cell membrane model.^38^ The details of preparation of erythrocyte ghosts can be found in the Experimental Section. Optical microscopy of the erythrocyte ghost solution revealed the presence of vesicle structures with a diameter of approximately 4 μm (Figure 5A). The presence of CD47 protein in the membrane of RBC-derived ghosts was also determined using the FITC-labeled anti-CD47 antibody as previously described by Mac et al.^39^ (Figure S23). The lipid content of the erythrocyte ghosts was 2.6 ± 0.3 mg/mL as determined by the Bligh–Dyer lipid extraction method.^40^ Thus, an aqueous solution of the HCP-MD100-0.27 polymer was added to the erythrocyte ghost suspension at a 2:1 HCP-to-lipid weight ratio and incubated for 3 days to investigate the possibility of forming ghost-derived fragments with HCPs. Both optical microscopy and cryo-TEM analyses of the solution after a 3 day incubation revealed the disappearance of the micron-sized vesicles and the formation of small nanostructures ∼8 nm in size (Figure 5B) as observed by ImageJ analysis of the cryo-TEM image. The nanostructures are presumed to consist of ghost membrane fragments stabilized by HCP. Our future efforts will be focused on the application of HCPs in the direct extraction of membrane proteins from whole cells or cell lysate.

## Conclusions

HCPs can induce the fragmentation of liposomes to form nanoscale HCP–lipid complexes in aqueous solutions. We have shown that the chain length and hydrophobic content are important parameters that modulate the interaction between liposomes and HCP polymers, resulting in varying extents and relative rates of liposome fragmentation. Long chain HCP polymers (DP_n_ = 100) with intermediate hydrophobicity (ca. 27 mol.% PNDG segment) most effectively induce liposome fragmentation while maintaining colloidal stability in water relative to the counterparts with shorter chain lengths or reduced hydrophobic contents. A high local hydrophobic contact between polymers and lipid membranes is postulated to be critical for efficient liposome fragmentation. HCP polymers can potentially be used for membrane protein extraction from the cell lysate or whole cell as a replacement for membrane scaffolding proteins, which will be the focus of future studies.

## Supporting Information Available

^1^H NMR spectra of Me-NCA, De-NCA, Bu-NCA monomers, and HCP polymers; SEC chromatogram; DLS analysis of polymer sizes in aqueous solutions; time-dependent DLS analysis of lipid–HCP complexes; cryo-TEM and negative stained TEM images of liposomes and HCP polymer solutions; cryo-TEM images of *E. coli* lipid–HCP complexes; and optical images of liposome solution, HCP solution, and HCP–lipid complex solution (PDF)
