# Supplementary material for: Uncovering the Optimal Molecular Characteristics of Hydrophobe-Containing Polypeptoids to Induce Liposome or Cell Membrane Fragmentation
Source: Biomacromolecules. 2023 Feb 21;24(3):1511–21. doi: 10.1021/acs.biomac.3c00028 (PMC10015453; doi:10.1021/acs.biomac.3c00028)
Supplement: Supplementary file 1 — bm3c00028_si_001.pdf [file bm3c00028_si_001.pdf]

Supporting Information

Uncovering the Optimal Molecular Characteristics of Hydrophobe-Containing Polypeptoids to Induce Liposome or Cell Membrane Fragmentation

Tianyi Yu,[a] Marzhana Omarova,[b] Meng Zhang,[a] Istiak Hossain,[b] Jianqiang Chen,[b] Omead Darvish,[a] Vijay T. John,[b,*] and Donghui Zhang[a,*]

[a.] *Department of Chemistry and Macromolecular Studies Group, Louisiana State University, Baton Rouge, LA 70803, United States*

[b.] *Department of Chemical and Biomolecular Engineering, Tulane University, New Orleans, LA 70118, United States*

Corresponds to: dhzhang@lsu.edu and vj@tulane.edu

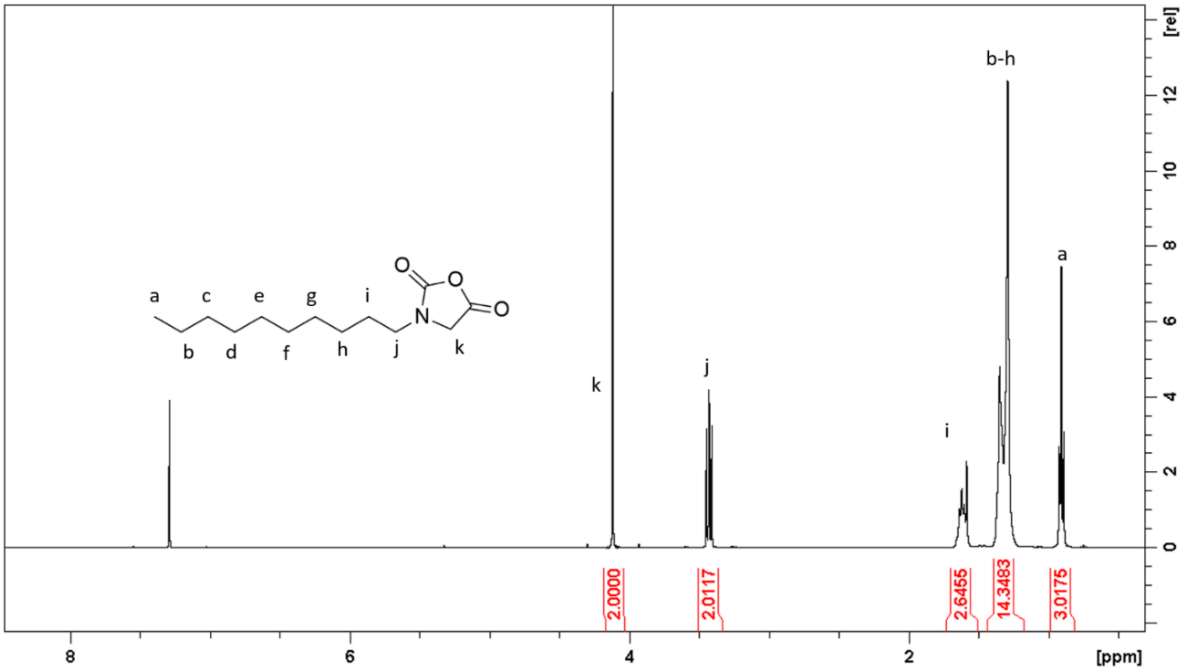

Figure S1. ^{1}H NMR spectrum of De-NCA monomer in $CDCl_3$.

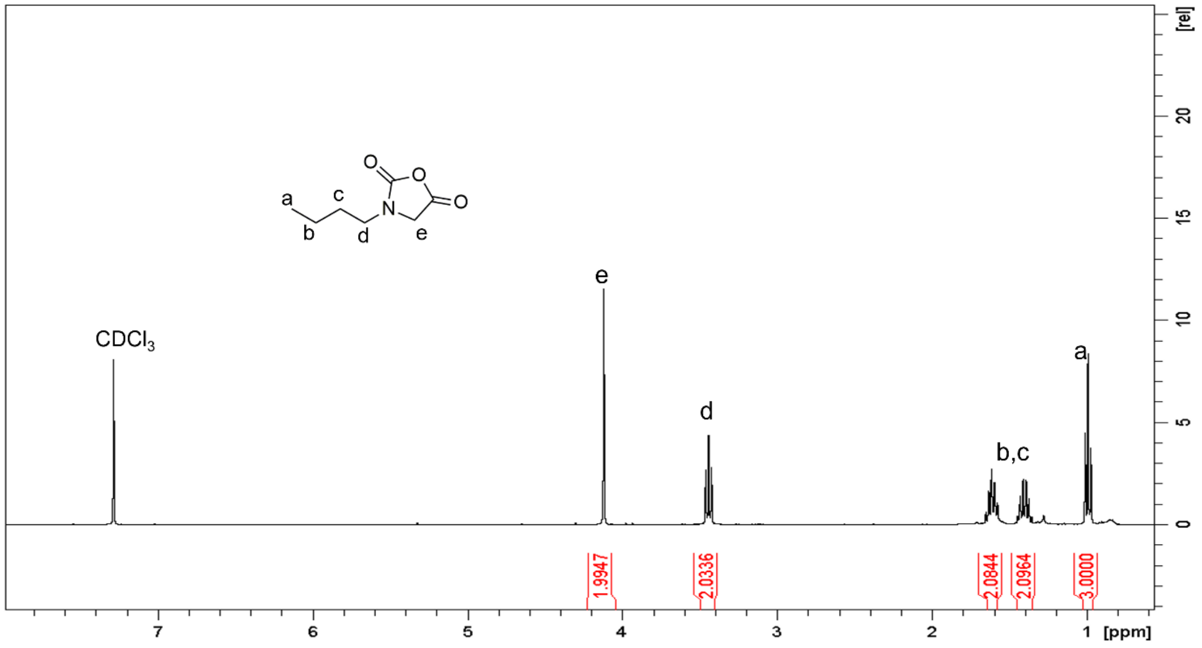

Figure S2. ^{1}H NMR spectrum of Bu-NCA monomer in $CDCl_3$.

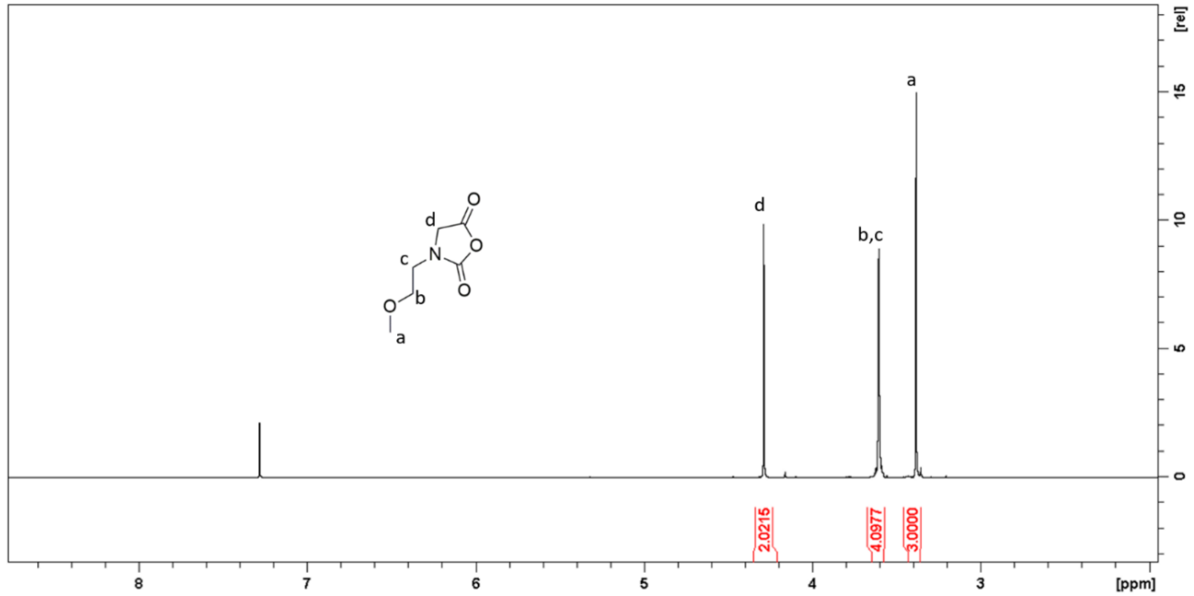

Figure S3. ^{1}H NMR spectrum of MeOEt-NCA monomer in $CDCl_3$.

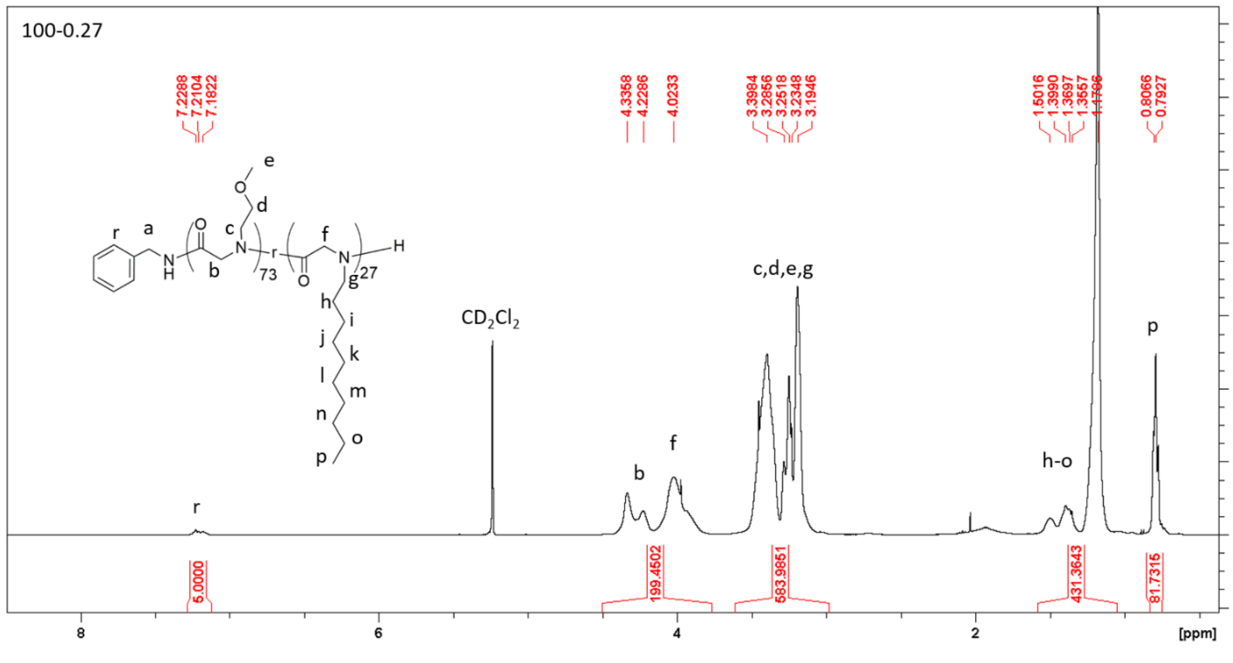

Figure S4. ^{1}H NMR spectrum of PNMeOEGt$_{73}$-*r*-PNDG$_{27}$ (HCP-MD100-0.27) random copolypeptoid in CD_2Cl_2.

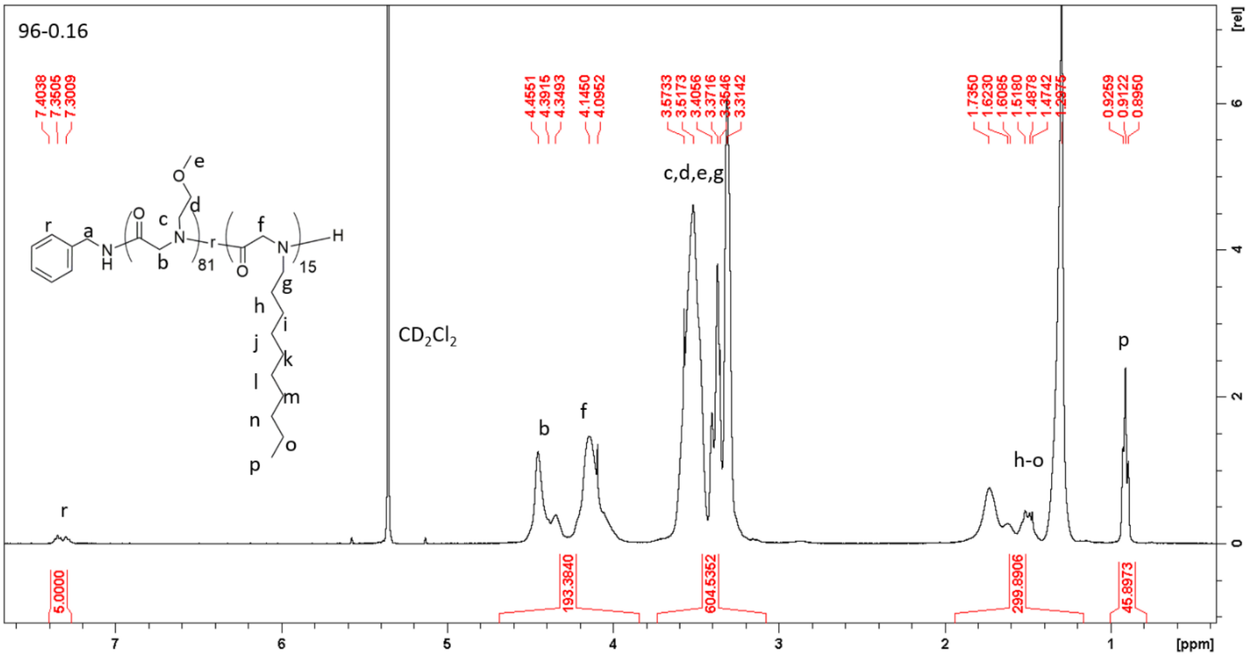

Figure S5. ^{1}H NMR spectrum of PNMeOEtG$_{81}$-*r*-PNDG$_{15}$ (HCP-MD96-0.16) random copolypeptoid in CD_2Cl_2.

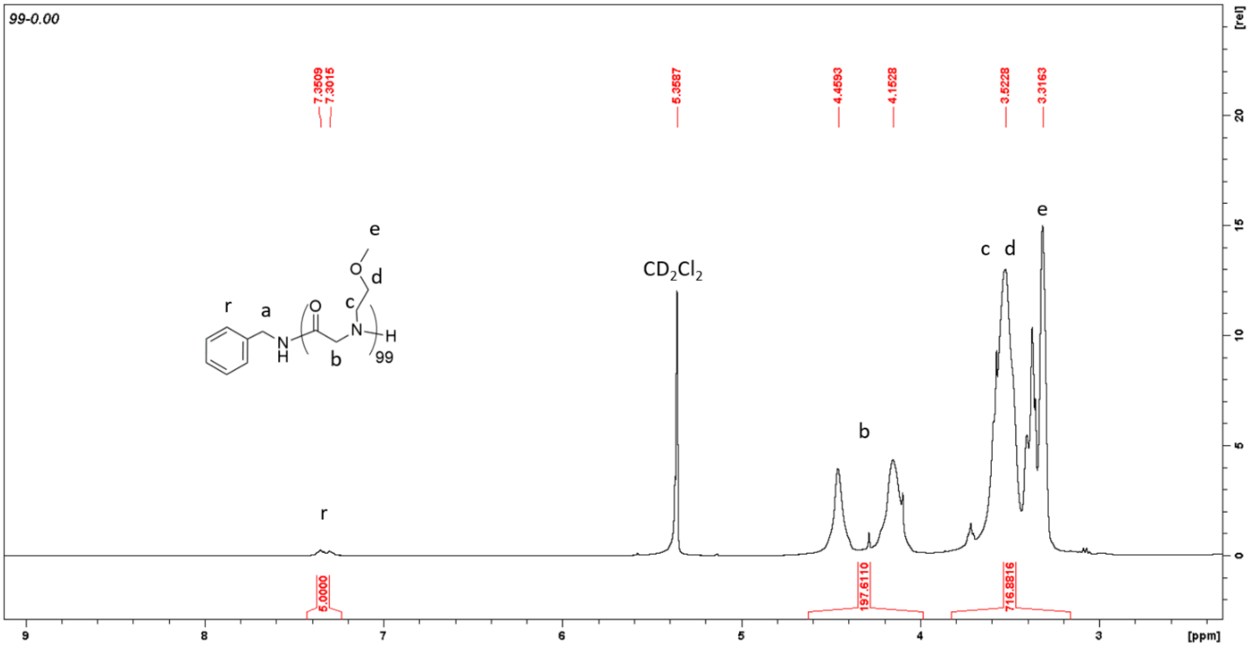

Figure S6. ^{1}H NMR spectrum of PNMeOEt$_{99}$ (NCP-M99-0.00) random copolypeptoid in CD_2Cl_2.

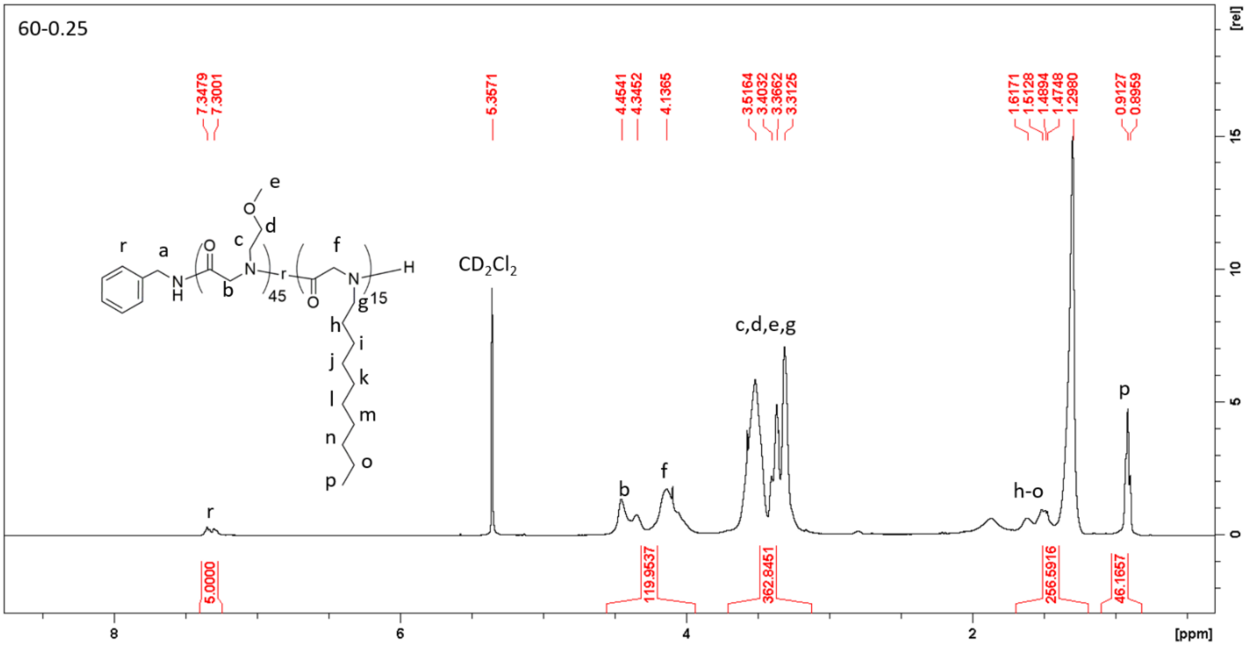

Figure S7. ^{1}H NMR spectrum of PNMeOEGt$_{45}$-*r*-PNDG$_{15}$ (HCP-MD60-0.25) random copolypeptoid in CD_2Cl_2.

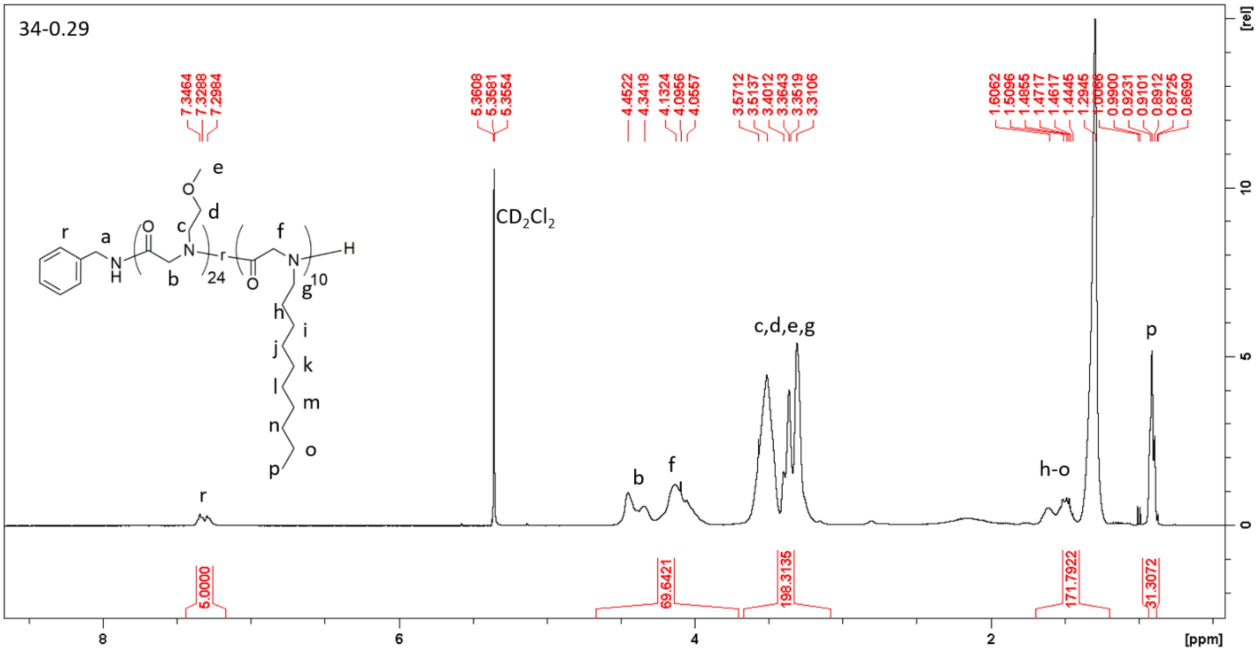

Figure S8. ^{1}H NMR spectrum of PNMeOEtG$_{24}$-*r*-PNDG$_{10}$ (HCP-MD34-0.29) random copolypeptoid in CD_2Cl_2.

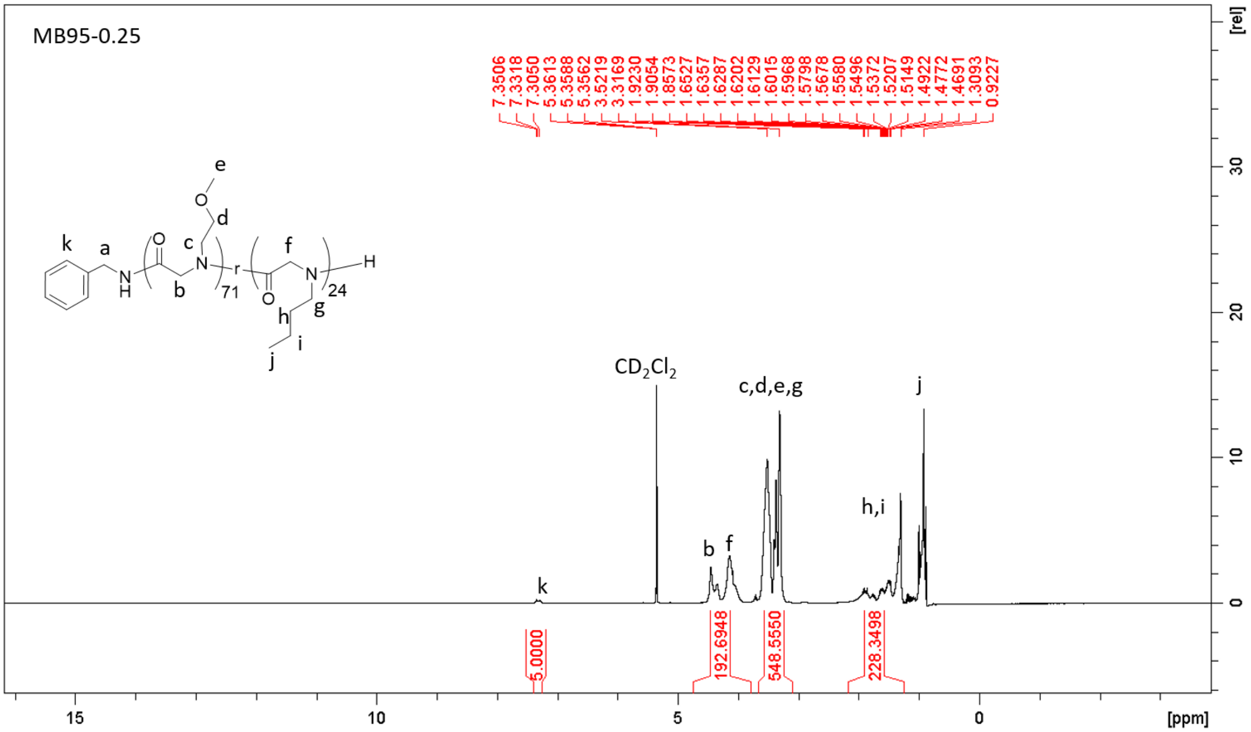

Figure S9. ^{1}H NMR spectrum of PNMeOEtG$_{71}$-*r*-PNBG$_{24}$ (HCP-MB95-0.25) random copolypeptoid in CD_2Cl_2.

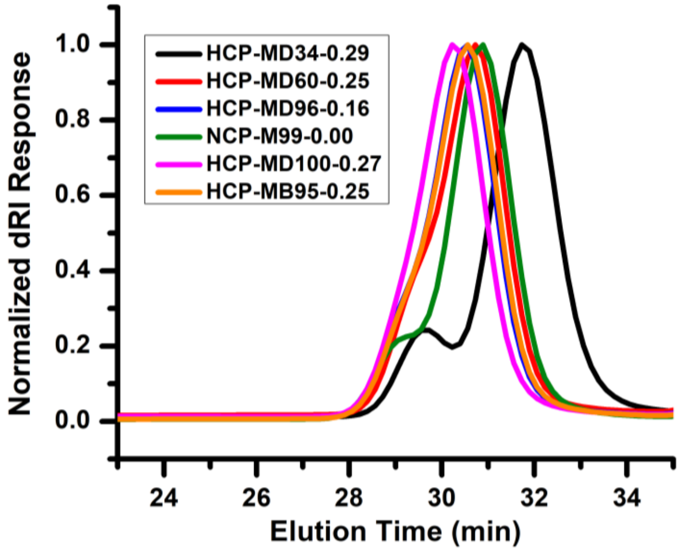

Figure S10. SEC-DRI chromatograms of HCP and NCP copolymers (Table 1) in HFIP with 3 mg/ml CF_3CO_2K at 40°C. All polymer except for the lowest molecular weight HCP-MD34-0.29 (Entry 2, Table 1) and NCP-M99-0.00 (Entry 6, Table 1) exhibited monomodal molecular weight distribution. The small shoulders appearing at the low elution time for these two samples are attributed to partial aggregation of the polymers in the HFIP/CF_3CO_2K (3 mg/ml) solvent.

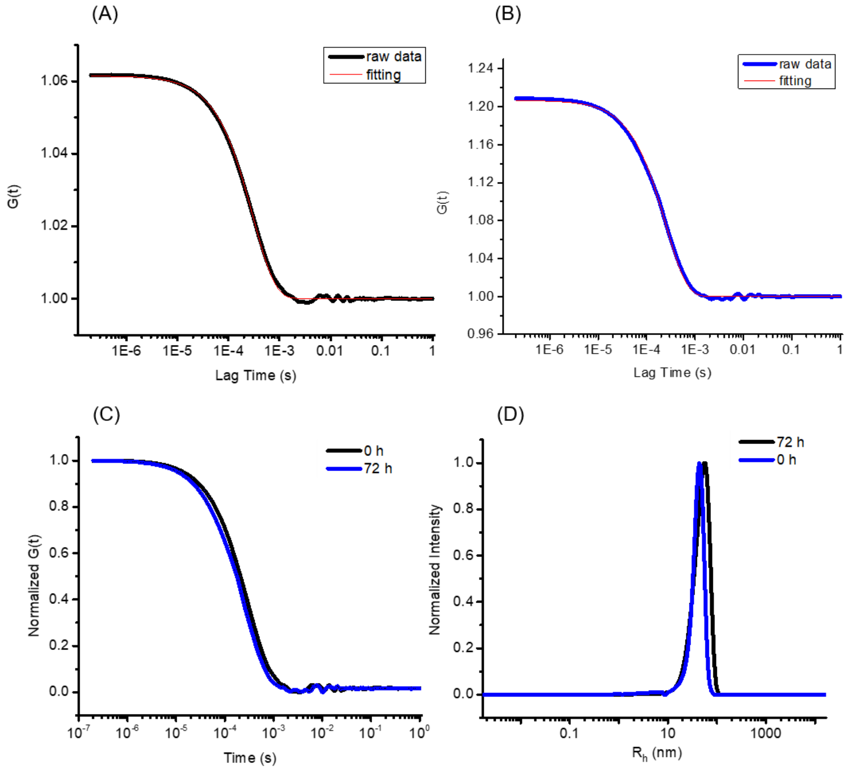

Figure S11. (A-B) Plots of autocorrelation function obtained by DLS measurements (black) with the fitting curve (red) using MEM method, (C) plots of normalized autocorrelation functions, and (b) the intensity-weighted particle size distribution for liposome solutions that was freshly prepared (R_h = 53.5 ± 0.3 nm) or allowed to stand at room temperature for 3 days (R_h = 45.1 ± 0.2 nm).

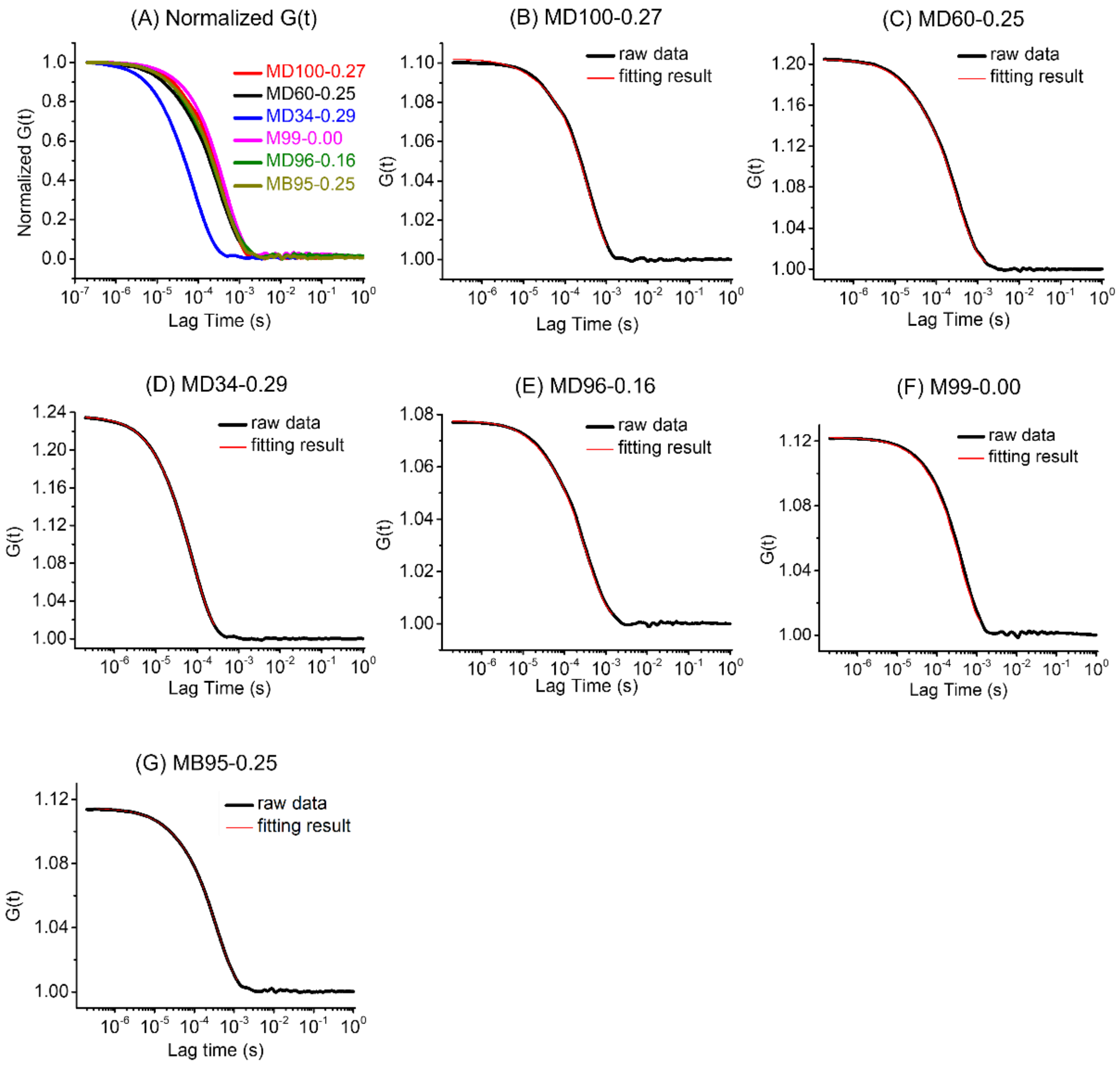

Figure S12. (A)-(E) Normalized autocorrelation function obtained by DLS measurements and the fitting curves using MEM method for the respective aqueous solutions of HCP-MD polymers with varying chain lengths (DP_n = 34, 60, 100) and varying hydrophobic content (PNDG mol.% = 16%, 27%), and (F) NCP polymer with DP_n = 99, and (G) MB polymer with DP_n = 95 and PNBG mol. % = 25% at 5 mg/ml. The coefficient of determination (R^2) for all the fittings are > 0.99.

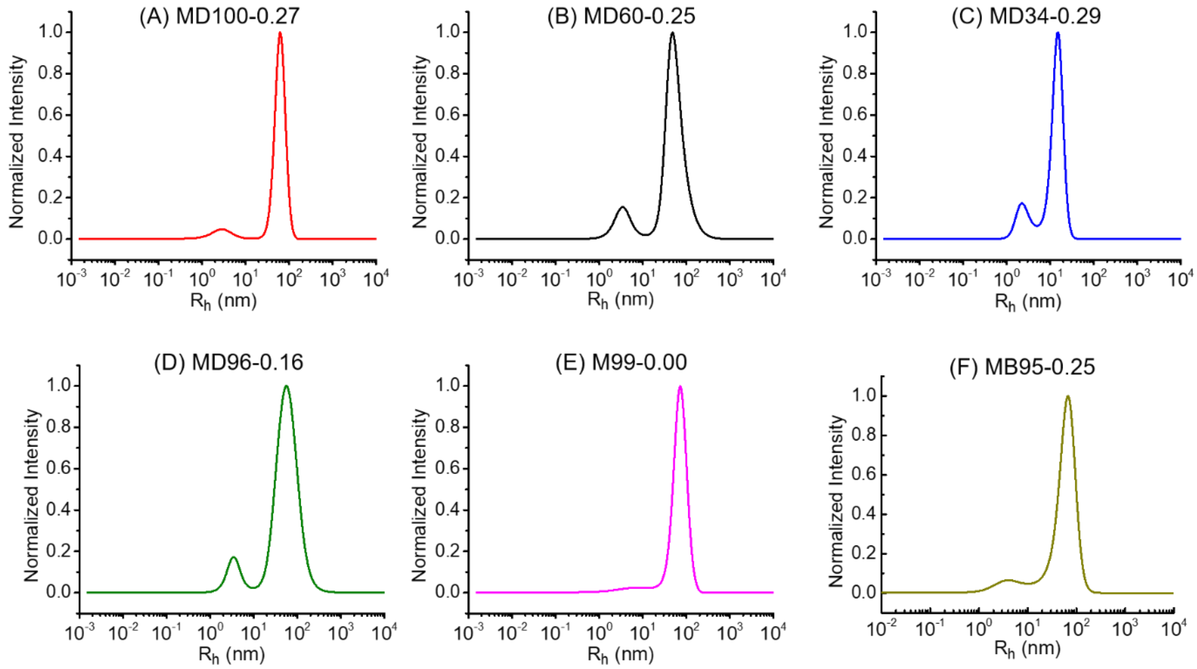

Figure S13. The intensity-weighted particle size distribution for (A)-(D) HCP-MD polymer solutions with varying chain lengths (DP_n = 34, 60, 100) and varying hydrophobic content (PNDG mol.% = 16%, 27%), and (E) NCP polymer solution with DP_n = 99 (M99-0.00), and (F) HCP-MB polymer solution with DP_n = 95 and PNBG mol. % = 25% (MB-95-0.25) at 5 mg/ml.

Table S1. Hydrodynamic sizes of H(N)CP polymers in aqueous solutions at 5 mg/ml concentration

H(N)CP Polymer [a]	$R_{h,1}$ (nm) [b]	$R_{h,2}$ (nm) [c]	N_1/N_2 [b,c]
MD100-0.27	3.6 ± 0.5	66.6 ± 0.3	1.53×10^5
MD60-0.25	4.1 ± 0.5	60.6 ± 0.4	1.03×10^5
MD34-0.29	2.8 ± 0.4	15.9 ± 0.3	1.35×10^3
MD96-0.16	4.9 ± 0.5	74.5 ± 0.5	3.08×10^5
M99-0.00	NA	78.0 ± 0.3	N/A
MB95-0.25	6.5 ± 0.7	73.1 ± 0.3	4.02×10^4

[a.] The sizes of HCP polymers were analyzed using MEM analysis method based on the results of DLS measurements; [b.] $R_{h,1}$ refers to the hydrodynamic radius corresponding to the intensity distribution peak at lower decay time, and N_1 represents the particle number of this population; [c.] $R_{h,2}$ refers to the hydrodynamic radius corresponding to the peak at higher decay time, and N_2 represents the particle number of this population.

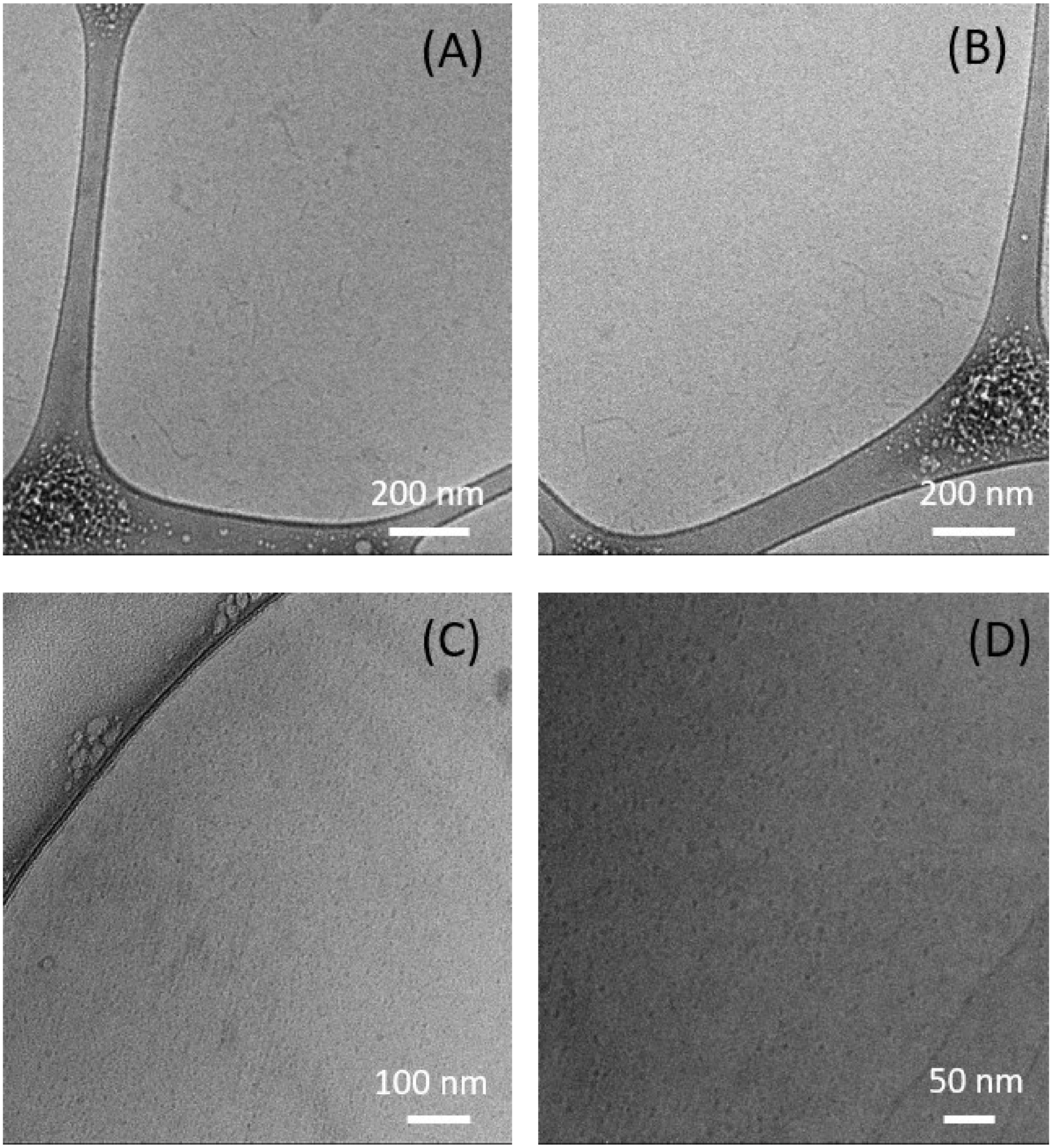

Figure S14. Cryo-TEM images of aqueous solutions of (A)-(B) HCP-MD100-0.27 and (C)-(D) HCP-MD34-0.29 polymers at 5 mg/ml concentration.

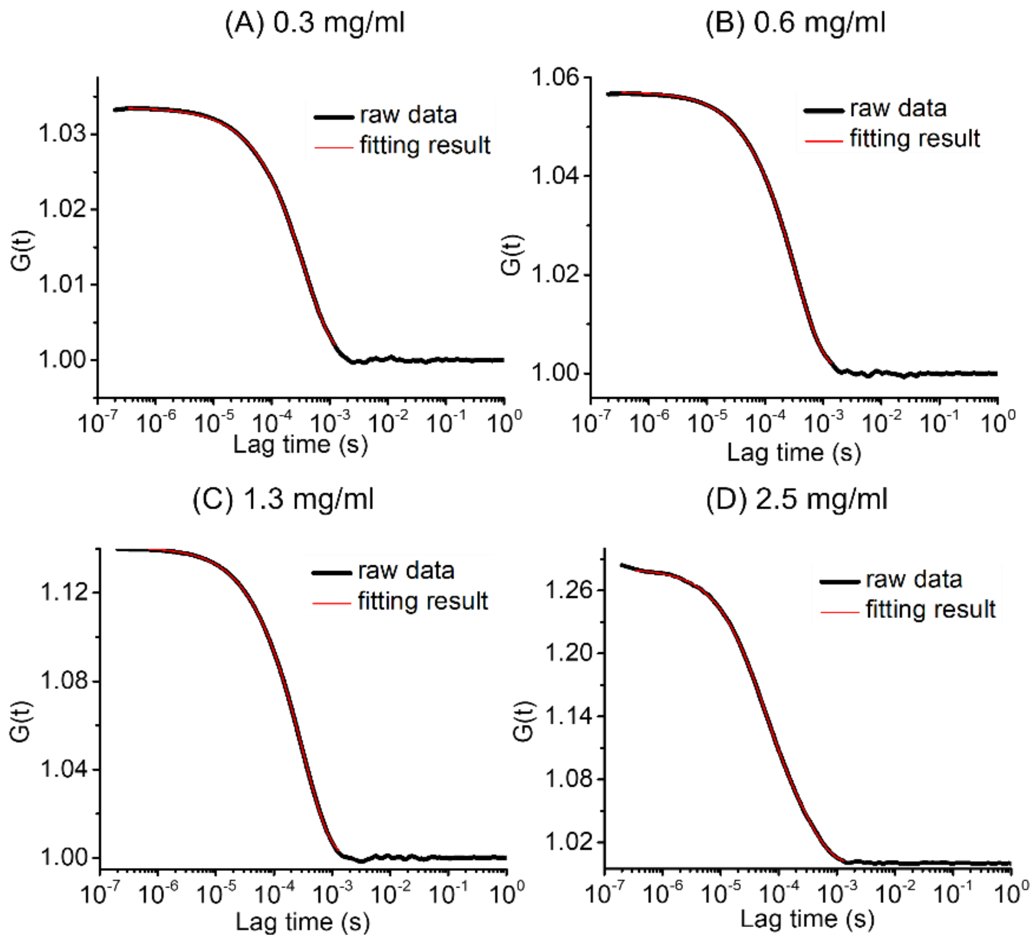

Figure S15. DLS autocorrelation function versus lag time (black) and MEM fitting curves (red) of liposome-HCP-MD100-0.27 solutions at polymer concentration of (A) 0.3 mg/ml, (B) 0.6 mg/ml, (C) 1.3 mg/ml, and (D) 2.5 mg/ml after incubating the solution at room temperature over 3 days. The coefficient of determination (R^2) for all the fittings are > 0.99.

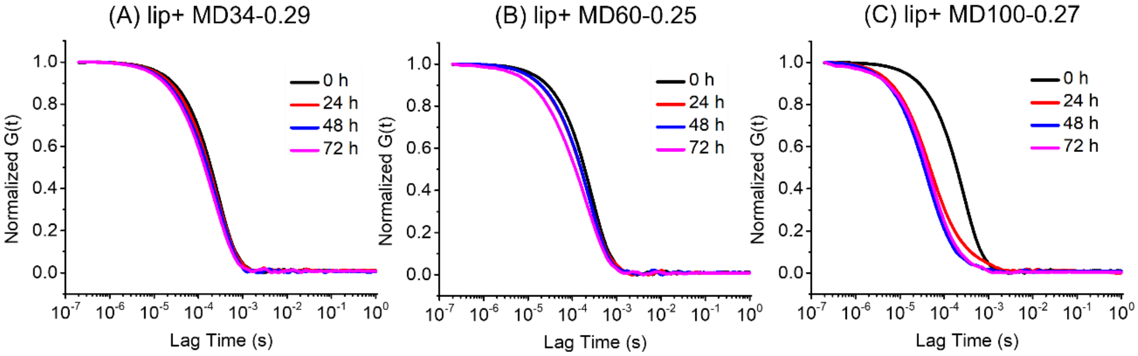

Figure S16. (A)-(C) DLS data showing the plots of normalized autocorrelation function versus lag time for the respective liposome-HCP polymer solution containing HCP-MD100-0.27, HCP-MD60-0.25 or HCP-MD34-0.29 during incubation at room temperature over the 3-days period.

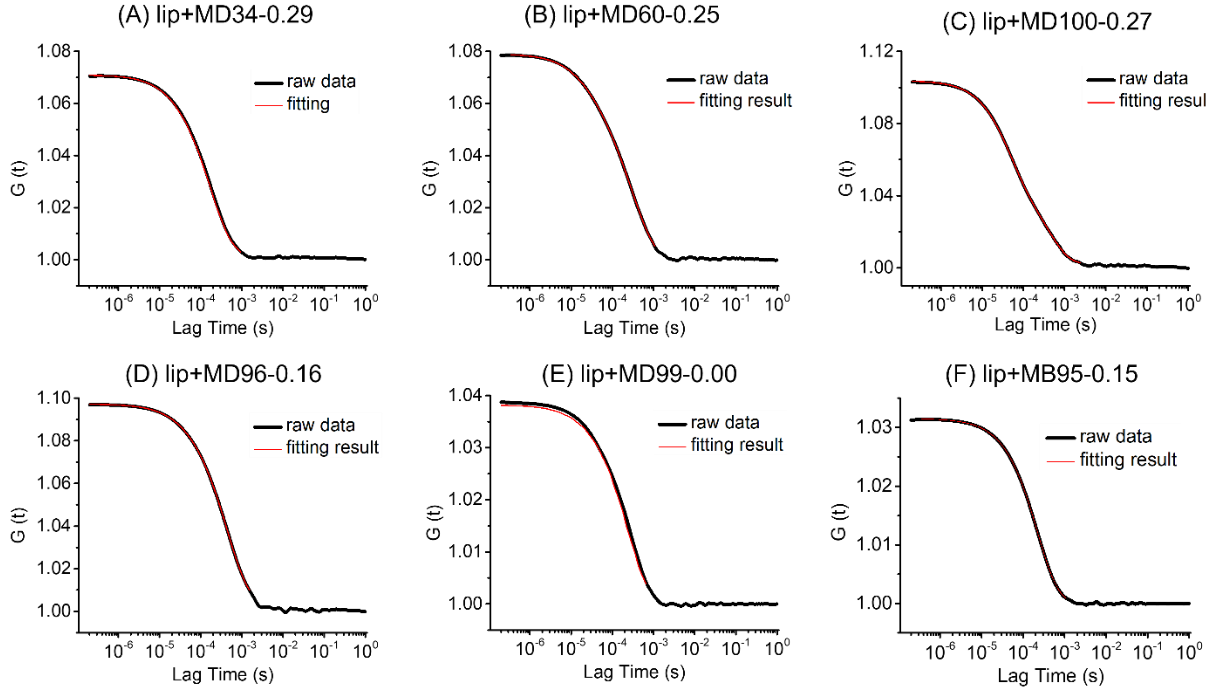

Figure S17. DLS autocorrelation function versus lag time (black) and MEM fitting curves (red) for the respective liposome-HCP polymer solutions containing (A) HCP-MD34-0.29, (B) HCP-MD60-0.25, (C) HCP-MD100-0.27, (D) HCP-MD96-0.16, (E) NCP99-0.00, and (F) HCP-MB95-0.25 after incubating at room temperature over 3 days. The coefficient of determination (R^2) for all the fittings are > 0.99.

Table S2. Hydrodynamic sizes of the lipid and HCP-MD100-0.27 complexes (lip+HCP-MD100-0.27) obtained at different polymer concentrations

HCP-MD100-0.27 concentration	$R_{h,1}$ (nm) [a]	$R_{h,2}$ (nm) [b]
0.3 mg/ml	NA	70.8 ± 0.4
0.6 mg/ml	7.8 ± 0.4	58.0 ± 0.3
1.3 mg/ml	12.7 ± 0.5	56.4 ± 0.2
2.5 mg/ml	9.3 ± 0.5	52.5 ± 0.2

[a.] The sizes of lipid and HCP-MD100-0.27 complexes (lip+HCP-MD100-0.27) were analyzed using MEM analysis method based on the results of DLS measurements. [b.] $R_{h,1}$ refers to the hydrodynamic radius corresponding to the intensity distribution peak at lower decay time. [c.] $R_{h,2}$ refers to the hydrodynamic radius corresponding to the peak at higher decay time.

Table S3. Hydrodynamic sizes of various lipid and H(N)CP complexes (lip+H(N)CP) obtained at 2.5 mg/mL polymer concentration

Lip+H(N)CP [a]	$R_{h,1}$ (nm) [b]	$R_{h,2}$ (nm)[c]
lip+HCP-MD100-0.27[a]	8.6 ± 0.4	77.4 ± 0.2
lip+HCP-MD60-0.25[a]	6.7 ± 0.4	73.6 ± 0.5
lip+HCP-MD34-0.29[a]	4.6 ± 0.5	34.7 ± 0.4
lip+HCP-MD96-0.16[a]	4.4 ± 0.2	43.0 ± 0.5
lip+NCP-M99-0.00[a]	N/A	75.2 ± 0.4
lip+HCP-MB95-0.25[b]	N/A	41.9 ± 0.4

[a.] The sizes of lipid and H(N)CP complexes (lip+HCP) were analyzed using MEM analysis method based on the results of DLS measurements.[b.] $R_{h,1}$ refers to the hydrodynamic radius corresponding to the intensity distribution peak at lower decay time. [c.] $R_{h,2}$ refers to the hydrodynamic radius corresponding to the peak at higher decay time.

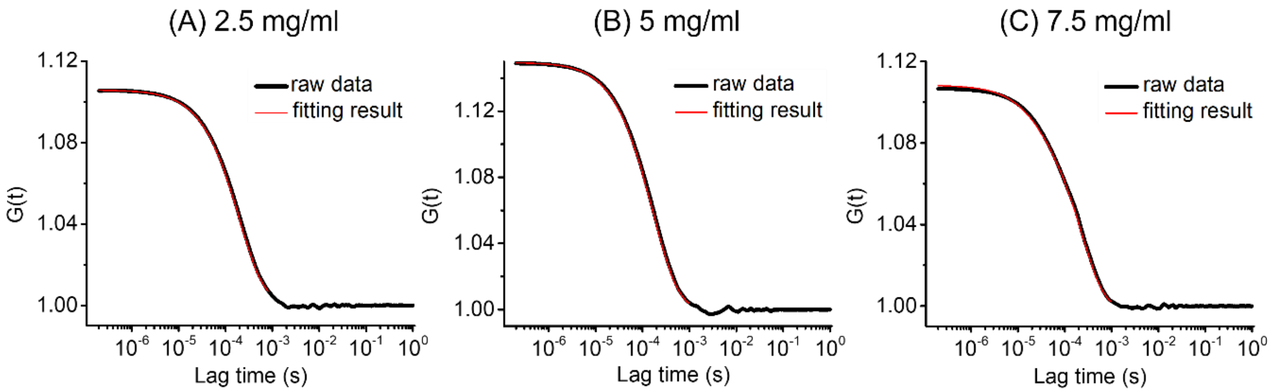

Figure S18. DLS autocorrelation function versus lag time (black) and MEM fitting curves (red) for the liposome-HCP-MD34-0.29 polymer solution with varying polymer concentration at (A) 2.5 mg/ml, (B) 5.0 mg/ml and (C) 7.5 mg/ml after incubation at room temperature for 3 days. The coefficient of determination (R^2) for all the fittings are > 0.99.

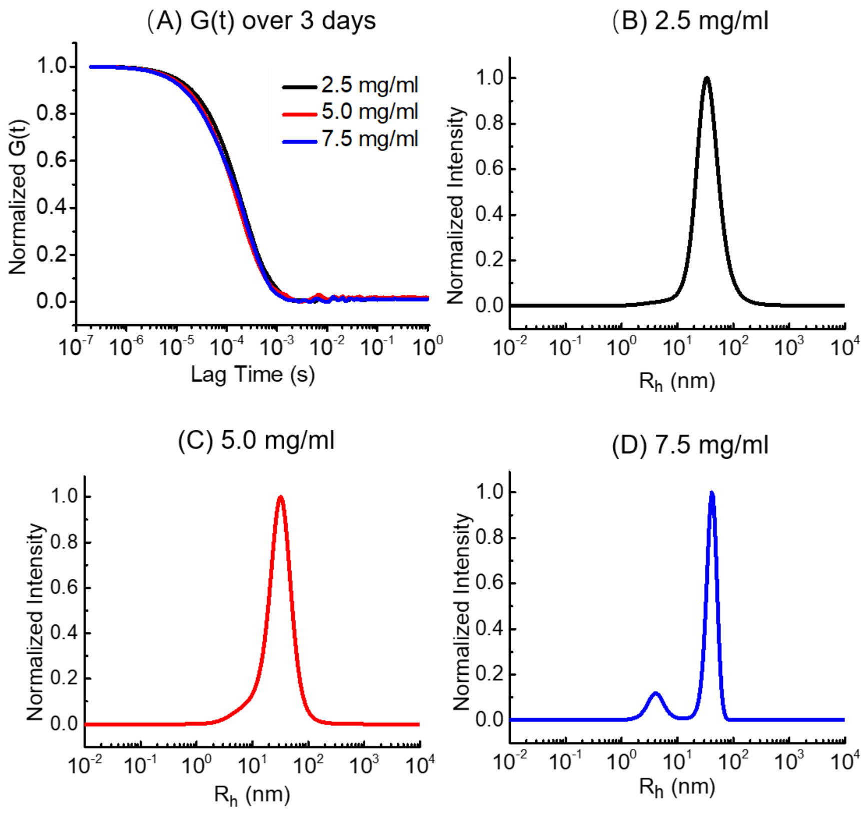

Figure S19. (A) Normalized DLS autocorrelation function for versus lag time and (B)-(D) the intensity-weighted particle size distribution for the liposome-HCP-MD34-0.29 polymer solution after mixing 2.5 mg/ml liposome aqueous solutions with (B) 5 mg/ml, (C) 10 mg/ml, and (D) 15 mg/ml of HCP-MD34-0.29 (v:v = 1:1) and incubated at room temperature for 3 days.

Table S4. Hydrodynamic sizes of lipid and HCP-MD34-0.29 complexes (lip+HCP-MD34-0.29) at varying polymer concentrations

concentration of HCP34-0.29	$R_{h,1}$ (nm) [a]	$R_{h,2}$ (nm) [b]
2.5 mg/ml	NA	42.1 ± 0.5
5.0 mg/ml	NA	36.4 ± 0.4
7.5 mg/ml	4.6 ± 0.3	42.8 ± 0.2

[a.] The sizes of HCP-MD34-0.29 and lipid complexes (lip+HCP34-0.29) were analyzed using MEM analysis method based on the results of DLS measurements. [b.] $R_{h,1}$ refers to the hydrodynamic radius corresponding to the intensity distribution peak at lower decay time. [c.] $R_{h,2}$ refers to the hydrodynamic radius corresponding to the peak at higher decay time.

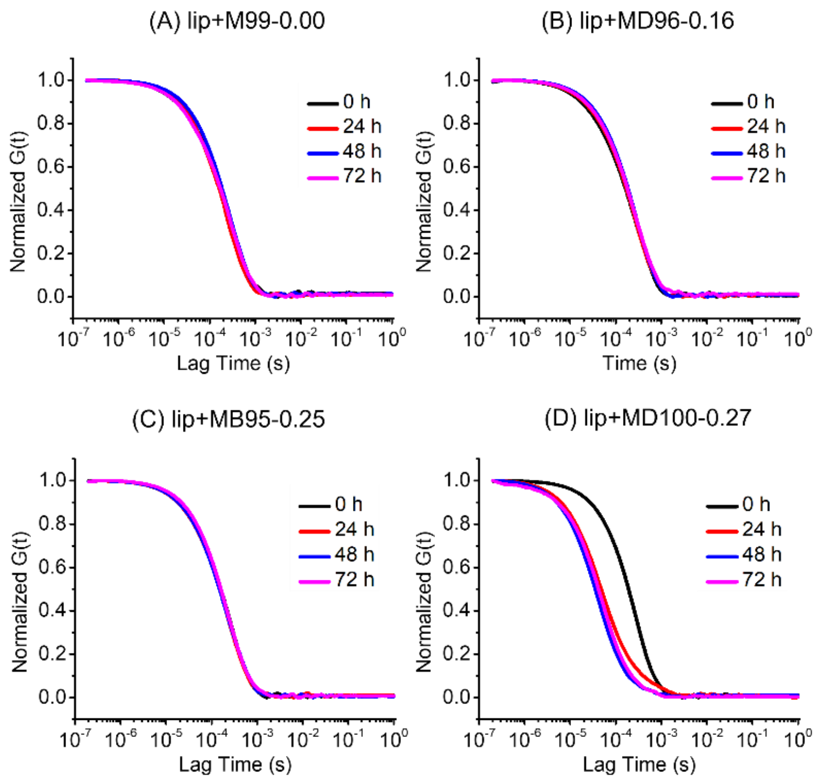

Figure S20. (A-D) DLS analysis showing the plots of the normalized autocorrelation function versus the lag time for the liposome-HCP polymer solutions containing HCP with varying hydrophobic content (*i.e.*, PNDG mol.% = 16% or 27%, PNBG mol. % = 25%) or the liposome-NCP polymer solution with NCP polymer (PNDG mol.% = 0%).

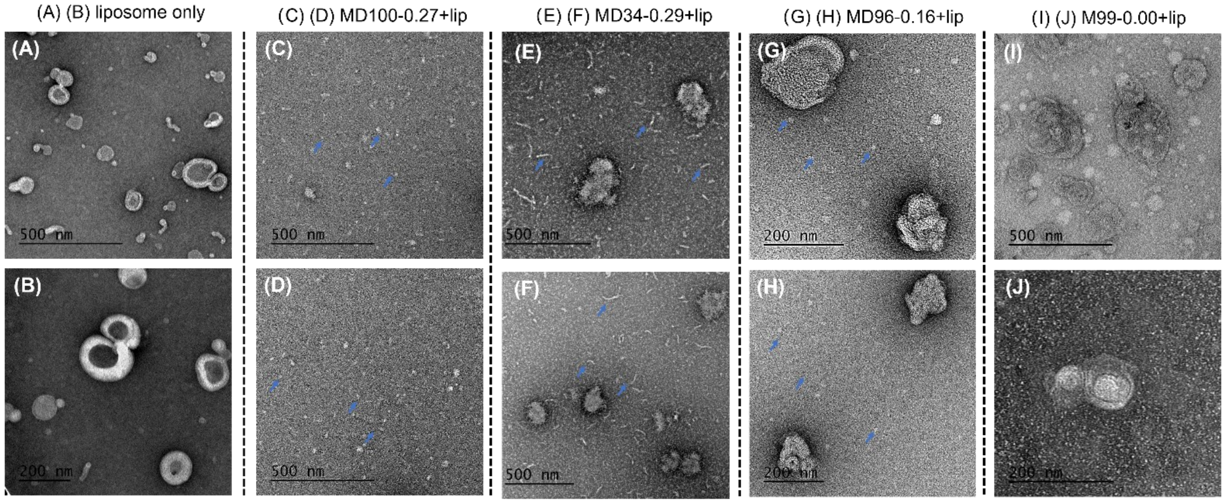

Figure S21. Representative negative stained TEM images of (A-B) liposome solution, (C-D) liposome-HCP-MD100-0.27 polymer solution, (E-F) liposome-HCP34-0.29 polymer solution, (G-H) liposome-HCP96-0.16 polymer solution, and (I-J) liposome-NCP99-0.00 polymer solution after incubation at room temperature for 3 days. The TEM samples were stained using uranyl acetate as a negative stain. The fragmented liposomes were indicated with blue arrows.

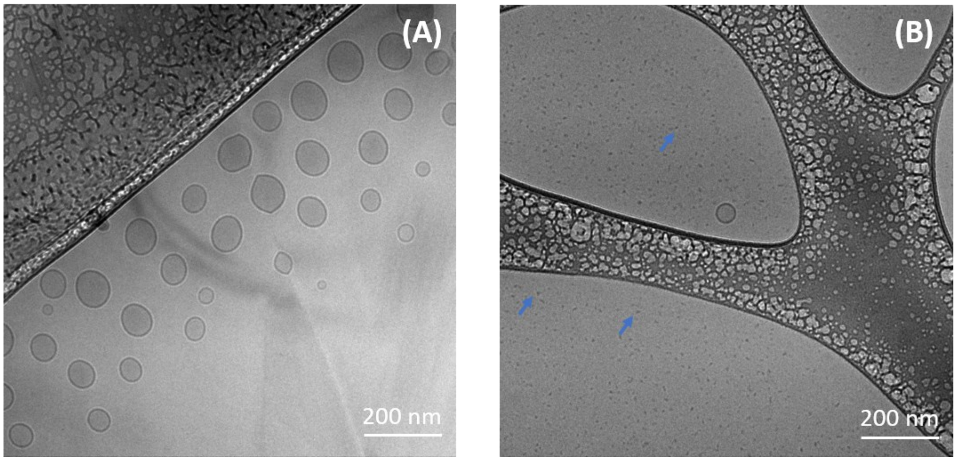

Figure S22. Cryo-TEM images of E. coli lipid derived liposomes (A) before and (B) after addition of HCP-MD92-0.22 polymers (fragmented liposomes indicated with arrows).

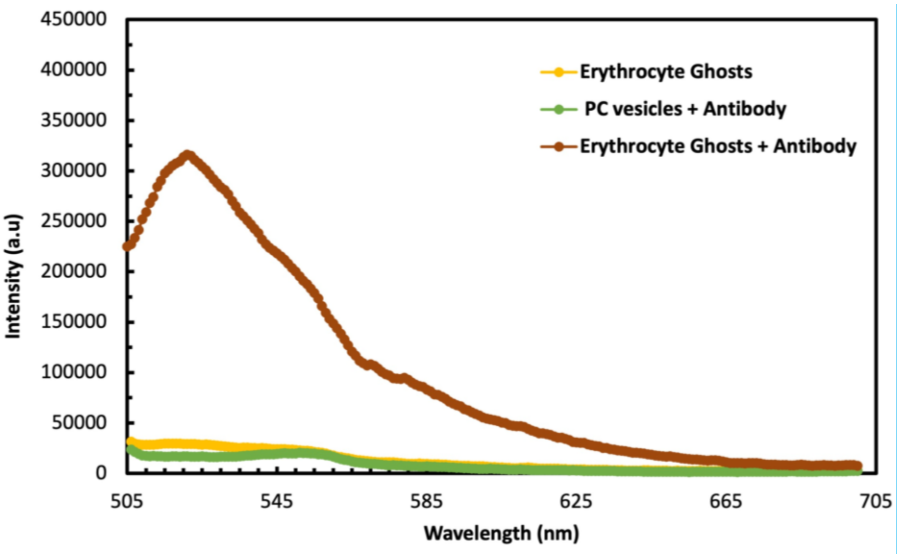

Figure S23. Fluorescence emission spectra of erythrocyte ghosts (background), erythrocyte ghosts conjugated with FITC-labeled anti-CD47, and PC vesicles (negative control). The fluorescence emission spectra were collected using an excitation wavelength of 465 nm. Erythrocyte ghosts upon incubation with the antibody exhibited a notable emission peak at 520 nm that is characteristics of FITC, supporting the presence of CD47. By contrast, PC vesicles upon incubation with the antibody did not exhibit any emission peak associated to FITC.

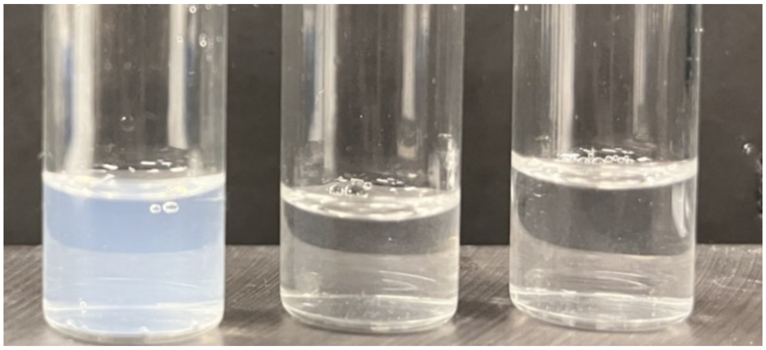

Figure S24. Photos showing the liposome solution (0.25 wt%) (left), HCP solution (0.5 wt%) (right), and HCP/lipid complex solution by mixing the respective liposome and HCP solutions at 1:1 (v:v) ratio (middle).
